# From disengagement to re-engagement: behavioral evidence for AI-driven adaptive tutoring interventions in intelligent tutoring systems

**DOI:** 10.3389/frai.2026.1936771

**Published:** 2026-10-29

**Authors:** Joselin García-Ortiz, Juan-Fernando Polanco

**Affiliations:** Escuela de Ingeniería en Ciberseguridad, Facultad de Ingenierías y Ciencias Aplicadas, Universidad de Las Américas, Quito, Ecuador

**Keywords:** adaptive learning, artificial intelligence in education, behavioral analytics, disengagement, educational data mining, hint-seeking, intelligent tutoring systems, learner engagement

## Abstract

Cognitive disengagement during intelligent tutoring system (ITS) interactions has been extensively characterized in terms of state detection and prevalence, yet the behavioral dynamics governing recovery from negative episodes remain poorly understood. This study analyzes cognitive-affective state dynamics in ASSISTments, a widely used mathematics ITS, using 597,242 interaction records from 799 secondary-level students collected over three academic years. A nine-phase analytical pipeline characterized state distribution, identified behavioral predictors of states through a three-stage feature selection procedure combining Kruskal–Wallis preselection, Random Forest importance ranking, and Recursive Feature Elimination with Cross-Validation, and modeled state transitions and recovery time from disengagement episodes toward the concentrating state. Results indicate that response time and hint-seeking behavior jointly account for 81.0% of Random Forest feature importance and exhibit large effect sizes in discriminating among states. Prevalence and severity were dissociated: boredom, the least frequent negative state, showed the lowest correct-response rate, while gaming, the most frequent, was also the most resistant to spontaneous recovery at the item level, though episodes that do exit resolve quickly given the brevity of individual gaming items. Recovery times differed substantially across negative states, ranging from a median of 18.0 s for gaming to 108.0 for boredom, roughly a six-fold difference. Average hint use during the negative episode emerged as the strongest behavioral predictor of fast recovery in the aggregate, with fast-recovering episodes showing 59% greater average hint use than slow-recovering episodes, although state-stratified analysis showed a positive association in frustration, a reversed association in confusion, a negligible association in gaming, and an initial positive association in boredom that reversed after filtering low-confidence assignments. These findings provide behavioral evidence for AI-driven adaptive interventions capable of promoting learner re-engagement and personalized support in Intelligent Tutoring Systems, shifting the focus from cognitive-state detection toward intervention strategies that sustain engagement.

## 1 Introduction

Sustained cognitive engagement is a key determinant of learning effectiveness: when students maintain active concentration, they process content more deeply, commit fewer errors, and consolidate knowledge more efficiently (Baker et al., 2010). Learning, however, rarely unfolds in a uniform state; students oscillate among cognitive-affective states that vary in quality, persistence, and impact on performance. Boredom, frustration, confusion, and gaming the system emerge regularly during learning and are associated with lower rates of correct response, reduced learning gains, and adverse long-term academic trajectories (Ocumpaugh et al., 2016). Intelligent tutoring systems (ITS) offer a privileged environment for studying these phenomena with precision. Unlike classroom-based observational studies, they generate continuous, timestamped interaction logs that systematically capture behavioral signals such as response times, hint requests, and error sequences, without requiring additional physical instrumentation (Baker et al., 2012; Rajendran et al., 2013; Rahman et al., 2024), making them natural laboratories for studying learning at ecological scale.

The technical capacity to detect cognitive-affective states from interaction logs has matured considerably, evolving from early models based on response patterns (Baker et al., 2004a) to multimodal systems that integrate facial, vocal, physiological, and interaction signals to trigger real-time adaptive interventions, with documented learning gains of up to 15 percentage points over static ITS (Kong et al., 2025). It has further been established that affective states exhibit differentiated temporal persistence patterns and that their impact on performance is quantifiable and substantial in both the short and long term (D'Mello and Graesser, 2011; Pardos et al., 2014). However, this evolution has kept the focus on detection accuracy rather than on understanding what occurs during and after the negative episode. The time students spend in unproductive states, the factors that determine the speed of recovery toward active cognitive engagement, and the role of student behavior during the episode itself have not been systematically addressed in the existing literature.

This gap carries both theoretical and practical implications. Theoretically, recovery dynamics constitute a phenomenon distinct from state detection and require analytical frameworks of their own, capable of capturing the full trajectory of the negative episode rather than only its point-in-time classification. Practically, designing effective adaptive interventions in ITS requires understanding which behaviors during the negative episode facilitate or delay the return to a productive state, information that current detection models do not provide. Such behavioral evidence is essential for developing AI-driven tutoring systems capable of personalizing support according to learners' evolving engagement, moving beyond state recognition toward timely and pedagogically meaningful intervention. This study analyzes cognitive-affective dynamics in ASSISTments, a secondary-level mathematics ITS widely used in educational research (Heffernan and Heffernan, 2014), drawing on 597,242 interaction records from 799 students. The central finding indicates that active hint-seeking during the negative episode is the strongest behavioral correlate of the return to cognitive engagement in the aggregate, though this association is not uniform across originating states, with direct implications for the design of AI-driven adaptive tutoring interventions that promote learner re-engagement and personalized learning support.

The dataset analyzed here predates the multimodal and generative ITS now emerging in the field, which raises the question of whether findings from a 2004 to 2007 deployment remain relevant to contemporary systems. The behavioral signals examined, namely response time, hint-seeking, and error patterns, are not artifacts specific to that interface generation: prior work using the same ASSISTments environment found that the structure of affective persistence held regardless of whether interactive pedagogical agents were present (Rodrigo et al., 2012), suggesting these signals index underlying self-regulatory and help-seeking processes rather than properties of a particular interface design. Moreover, the sensor-free affect detector underlying this dataset has continued to serve as a reference point well beyond its original deployment: subsequent work refined the original detector architecture using deep learning (Botelho et al., 2017), and detectors built on the same class of interaction logs continue to be applied, largely unchanged in their underlying logic, to more recent ASSISTments cohorts and downstream tasks such as knowledge tracing. This sustained reuse indicates that the behavioral constructs captured by these signals remain a live reference point in the field, even though the specific numeric estimates reported here should be validated on contemporary, AI-driven ITS platforms before being assumed to transfer directly.

The behavioral variables prioritized in this analysis are grounded in the self-regulated learning literature on help-seeking, which frames requesting assistance as an adaptive strategy learners deploy when self-monitoring signals that independent progress has stalled (Newman, 1994). Under this framework, hint requests, response latency, and error accumulation are not arbitrary log fields but observable traces of the underlying regulatory decision to seek external support rather than persist unassisted, a distinction that has informed help-seeking research in intelligent tutoring systems specifically (Aleven et al., 2006). This theoretical grounding motivates treating hint-seeking as a candidate mechanism for recovery from disengagement, while also cautioning against interpreting its association with recovery speed as evidence of a single adaptive function: the same surface behavior can reflect either adaptive help-seeking or its maladaptive counterpart, since excessive or indiscriminate help requests are also a recognized marker of avoidance-oriented and dependency-oriented help-seeking profiles (Karabenick and Newman, 2009).

While detection-focused studies have mapped the prevalence and persistence of cognitive-affective states (Baker et al., 2010) and chronometric work has quantified how long students remain within a given state (Botelho et al., 2018), neither line of research has examined the trajectory *out* of a negative episode or identified which behaviors accelerate that exit. Intervention studies targeting hint-seeking, in turn, have manipulated access to scaffolding to measure its effect on subsequent learning (Maniktala et al., 2020; Vanacore et al., 2024), but have not examined hint use as a naturally occurring behavioral signal of recovery from an ongoing disengagement episode. This study differs from both lines of work by treating recovery time itself as the outcome of interest and by identifying, from unmanipulated interaction logs, which behaviors exhibited *during* the negative episode, rather than the episode's frequency, duration, or an experimentally imposed scaffolding condition, predict how quickly a student returns to productive engagement.

Four research questions guide the analysis. The first examines how cognitive-affective states are distributed across interactions and to what extent they exhibit temporal self-persistence (RQ1). The second investigates which behavioral variables significantly discriminate among states, and with what effect size beyond statistical significance (RQ2). The third examines how long recovery takes from negative episodes toward concentration, and whether this time differs by originating state (RQ3). The fourth identifies which behavioral factors during the negative episode predict faster recovery, and what implications these carry for the design of adaptive intervention strategies in ITS (RQ4).

The main contributions of this work are as follows:

- A large-scale characterization of cognitive-state recovery in an ITS. We measure how long students remain disengaged, and how that duration changes depending on the state they started from.
- A shift in focus: from detecting the state, to evaluating behavior during the negative episode. We identify which specific student behaviors predict a faster return to a productive state.
- A three-stage feature selection procedure, combining statistical preselection, Random Forest importance, and Recursive Feature Elimination with Cross-Validation (RFECV). This procedure was applied together with Markov transition modeling and recovery-time analysis, all within a single reproducible pipeline.
- Design-relevant evidence: hint-seeking during disengagement is associated with faster recovery in frustration, but not consistently in boredom, confusion, or gaming. This has direct implications for how adaptive interventions should be prioritized in an ITS.

## 2 Related work

Cognitive-affective states in ITS have been extensively documented as determinants of academic performance and learning trajectories. Baker et al. (2010) characterized the distribution and persistence of six states across three different ITS with populations from the United States and the Philippines, establishing that engaged concentration was the most prevalent state, boredom the most persistent and most detrimental to learning, and that frustration was less harmful than prior hypotheses suggested. Rodrigo et al. (2012) confirmed that these persistence dynamics held regardless of the presence of interactive pedagogical agents, suggesting that the structure of affective transitions is a property of the student and the content rather than of the system's surface-level design. D'Mello and Graesser (2011) modeled the temporal dimension of these states through exponential decay rates, proposing a tripartite classification of persistent, transitory, and intermediate states that became the predominant reference framework for the following decade. McQuiggan et al. (2010) documented that empathetic responses from pedagogical agents modified transition probabilities between states in narrative environments, evidencing that affective dynamics are sensitive to the design of system interventions. Andres et al. (2019) extended this analysis to the Betty's Brain environment, arguing that existing models did not fully capture the frequency or meaning of state transitions, and that boredom functioned as an indicator of student knowledge level rather than as an independent affective state.

Automatic detection of cognitive-affective states from interaction logs has been one of the most productive lines of research in the field. Baker et al. (2004b) identified gaming the system as behavior aimed at exploiting the system's regularities without engaging with the content, documenting that its frequency correlated with posttest scores to a magnitude comparable to that of prior domain knowledge. Baker et al. (2004a) introduced the Latent Response Model to automatically detect gaming from response patterns, laying the groundwork for sensor-free detection. Baker et al. (2012) demonstrated that models trained exclusively on students' semantic actions could detect four affective states with above-chance accuracy under strict validation, establishing the feasibility of large-scale detection from existing interaction logs. Rajendran et al. (2013) complemented this approach with a theory-driven model to predict frustration in Mindspark, a large-scale mathematics ITS deployed in India, validated through independent human observation. Rahman et al. (2024) advanced toward cross-platform generalization, using Bayesian classifiers to identify a minimal, generalizable subset of features for frustration detection across distinct educational systems. Pardos et al. (2014) extended the scope of these detectors by demonstrating that affective states estimated throughout the school year predicted performance on high-stakes standardized tests, consolidating the predictive validity of detection models beyond the immediate context of the interaction.

The most recent developments in state detection point toward multimodal systems that integrate signals of diverse nature to improve accuracy and real-time intervention capacity. Hasnine et al. (2023) proposed a learning analytics system that detects engagement states from facial expression data in online environments. Kong et al. (2025) presented a framework that integrates facial, vocal, physiological, and interaction signals to estimate states and select adaptive interventions, reporting learning gains of approximately 15 percentage points over a static ITS in a controlled evaluation. These systems represent a substantial advance in real-time responsiveness, although their implementation requires sensing infrastructure that is not always available in real educational settings. This evolution has consolidated the technical capacity to identify the moment of disengagement with increasing precision, but it keeps the focus on state classification rather than on the recovery dynamics that follow the negative episode.

Hint-seeking constitutes the primary scaffolding mechanism in problem-solving ITS and has received growing attention as a behavioral variable informative of cognitive state. Maniktala et al. (2020) documented that embedding hints directly within the student's workspace significantly increased their use and produced efficiency gains for students with low prior knowledge. Price et al. (2017) found that the quality of the first hints received on a task was positively associated with future help-seeking, suggesting that initial experience with scaffolding shapes subsequent request patterns. Schulz and Voermanek (2025) showed that hint abuse negatively mediated the effect of help-seeking on learning, but only for step-by-step hints and not for strategic hints, pointing to a functional distinction between types of scaffolding. Schulze Bernd and Chounta (2024) found that task difficulty, student age, and attitude toward hints moderated the relationship between help-seeking and cognitive state, suggesting that the value of scaffolding is context-dependent. Liu et al. (2025) documented that hint-request rate was negatively associated with problem-level learning in a chemistry ITS, reinforcing the distinction between functional and evasive use of scaffolding.

Studies conducted specifically within ASSISTments are of particular relevance for situating the contribution of this analysis. Walonoski and Heffernan (2006) were the first to analyze gaming on that platform through automatic detection, confirming the generality of the phenomenon in an environment structurally distinct from Cognitive Tutor. Paquette and Baker (2017) compared gaming patterns between ASSISTments and two versions of Cognitive Tutor, finding that the differences were more attributable to the environment than to the student population, a result implying that findings derived from ASSISTments require explicit validation before being generalized. Ocumpaugh et al. (2016) linked affective engagement indicators extracted from ASSISTments to STEM vocational trajectories in higher education, documenting that confusion and engaged concentration had differential effects on long-term self-efficacy.

Lu et al. (2024) analyzed scaffolding use in ASSISTments using deep neural networks, finding that appropriate hint use produced modest growth in mathematical skills, whereas excessive use was counterproductive due to over-reliance on the system. Vanacore et al. (2024) used a randomized controlled trial to evaluate the effect of restricting hint access for students prone to gaming in ASSISTments, finding benefits from delayed access, though without fully eliminating the associated learning disadvantage. Together, these two studies establish that hint use in ASSISTments has differential effects depending on the student's behavioral profile and the type of scaffolding access, underscoring the need for models that capture the function of hint-seeking behavior in context rather than its absolute frequency. Taken together, this body of work establishes a solid foundation on detection, prevalence, and scaffolding use in ASSISTments, but none has operationalized cognitive recovery from sustained negative episodes or identified which behaviors during those episodes predict a faster return to the concentrating state, which is the gap that defines this study's contribution to the ITS literature.

Table 1 situates this study against the most relevant prior work, comparing four aspects: whether each study detected cognitive states, modeled temporal transitions, addressed learner re-engagement, or identified behavioral predictors of recovery specifically.

**Table 1:** Positioning of this study relative to closely related prior work.

Study	State detection	Transition modeling	Recovery analysis	Behavioral predictors of recovery
Baker et al. (2010)	✓	✓	—	—
Pardos et al. (2014)	✓	—	—	—
Botelho et al. (2018)	✓	—	Duration only	—
Maniktala et al. (2020)	—	—	—	Hint access (experimental)
Vanacore et al. (2024)	—	—	—	Hint access (experimental)
This study	✓	✓	✓	✓(observational)

## 3 Materials and methods

Figure 1 illustrates the complete architecture of the implemented analytical pipeline, from raw interaction logs in the ASSISTments system to the identification of behavioral predictors of engagement recovery. The pipeline is organized into five functional blocks, executed across nine sequential phases: data preparation and cognitive state construction, feature selection, statistical validation, temporal dynamics modeling, and state duration and engagement recovery analysis.

**Figure 1:**
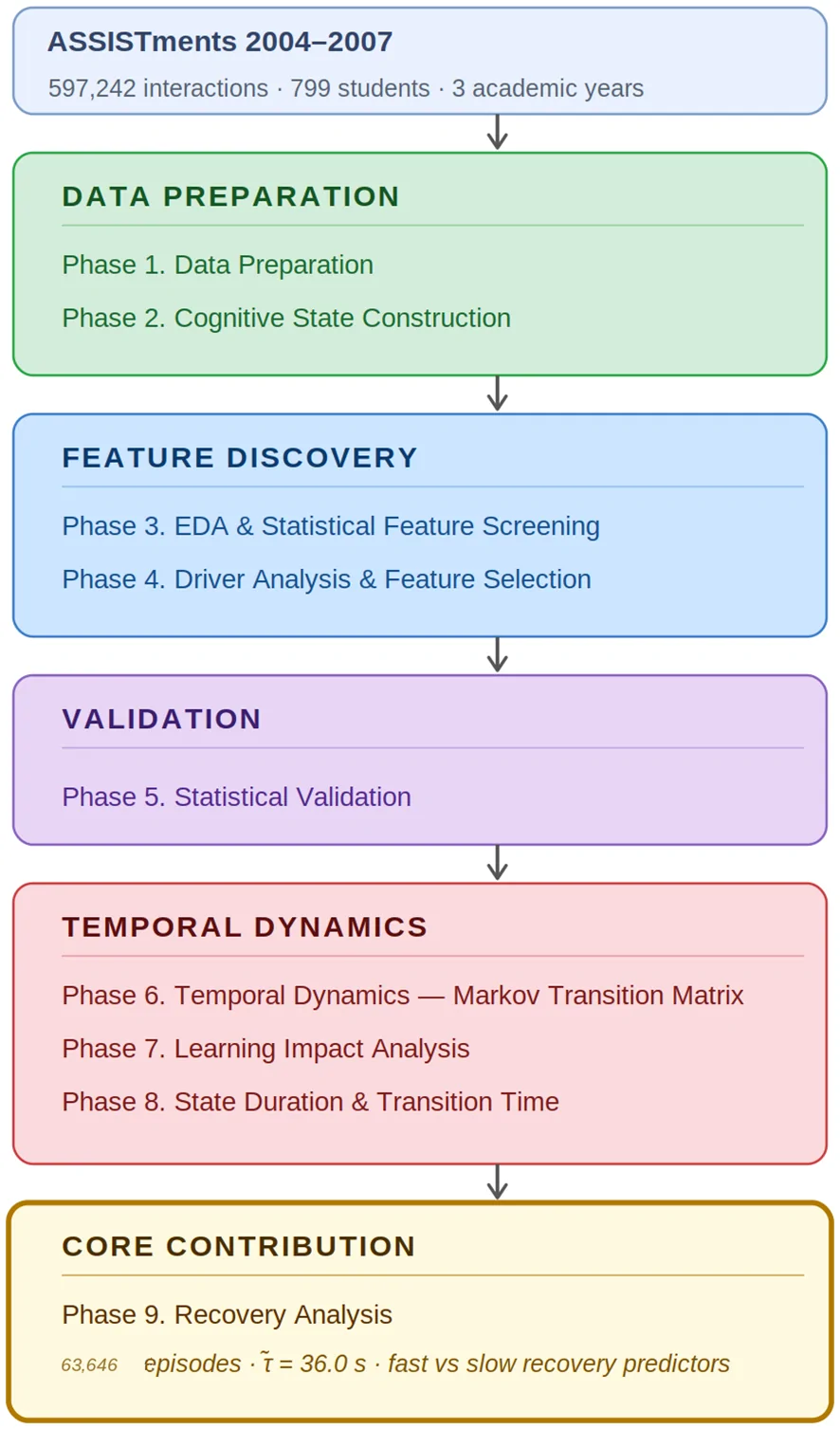
Nine-phase analytical pipeline for cognitive-affective analysis in ASSISTments.

Several of these phases additionally include a targeted robustness check: assignment-margin ambiguity, detector circularity, non-independence of observations, and the order of the Markov model. Each check is described alongside the corresponding phase in the following subsections.

### 3.1 Dataset and preprocessing

The dataset comprises 597,242 interaction records from 799 secondary-level students, collected between September 2004 and May 2007 across three consecutive academic years. Each record corresponds to an item solved by a student and includes Unix-format timestamps, performance variables, task-behavior variables, cumulative error history, and six probability scores estimated by the affect detector embedded in the system. This detector was trained in prior work using field observations collected under the BROMP protocol (Ocumpaugh et al., 2015; Baker et al., 2020); no BROMP field observations were conducted as part of the present study, which relies exclusively on the pre-existing, already-labeled interaction dataset. Of the available variables, 15 were selected, organized into four conceptual categories described in Table 2.

**Table 2:** Selected variables for analysis, by category.

Category	Variables
Identity and time	ITEST_id, startTime
Performance	correct, attemptCount
Task behavior	timeTaken, hintCount, consecutiveErrorsInRow
Error history	frPast5WrongCount, frPast8WrongCount, totalFrTimeOnSkill
Cognitive-affective states	RES_BORED, RES_CONCENTRATING, RES_CONFUSED, RES_FRUSTRATED, RES_OFFTASK, RES_GAMING

Timestamps were converted to datetime objects, and records were sorted chronologically within each student to preserve the temporal structure of interaction sequences. Preprocessing then verified dataset integrity prior to any transformation: the cognitive-affective scores (RES_) are complete estimates produced by the embedded detector for all interactions, and the behavioral and error-history numeric variables likewise showed no missing values. The dataset was complete from initial loading, with no missing values across any of the 15 selected variables, so neither imputation nor record deletion was necessary at this stage.

### 3.2 Primary cognitive state assignment

ASSISTments incorporates a cognitive-affective state detector trained on field observations collected using the BROMP protocol (Ocumpaugh et al., 2015; Baker et al., 2020). For each recorded interaction, the detector produces a vector of six continuous probabilities representing the estimated likelihood that the student is in each of the following states: RES_BORED, RES_CONCENTRATING, RES_CONFUSED, RES_FRUSTRATED, RES_OFFTASK, and RES_GAMING. Since the analysis requires a discrete label per interaction for sequence modeling, an argmax rule was applied to this vector, selecting the state with the highest estimated probability as the primary cognitive state. This procedure is standard in the sensor-free affect detection literature for ITS (Baker et al., 2010) (Equation 1):


$$\begin{array}{l} s_{i}=\arg\underset{c\in C}{\max}p\left(c|x_{i}\right) \end{array}$$ (1)

where C = {*BORED, CONCENTRATING, CONFUSED, FRUSTRATED, OFFTASK, GAMING*} and *p*(*c*∣*x*_*i*_) is the probability estimated by the detector for state *c* at interaction *i*.

Two assignment-quality metrics were additionally computed. Detector confidence is defined as the maximum value of the probability vector and reflects the certainty of the resulting assignment (Equation 2):


$$\begin{array}{l} {\text{conf}}_{i}=\underset{c\in C}{\max}p\left(c|x_{i}\right) \end{array}$$ (2)

The assignment margin is defined as the difference between the probability of the assigned primary state and that of the second most probable state, and indicates the degree of separation between the dominant state and its closest alternative (Equation 3):


$$\begin{array}{l} {\text{margin}}_{i}=p\left(s_{i}|x_{i}\right)-\underset{c\neq s_{i}}{\max}p\left(c|x_{i}\right) \end{array}$$ (3)

Both metrics were retained as covariates for subsequent analyses, following the use of assignment-quality metrics documented in previous studies using this same dataset (Botelho et al., 2018). Interactions with a margin below 0.10 are considered potentially ambiguous assignments and are reported as an indicator of the uncertainty inherent to the detection process, following the criterion proposed by Baker et al. (2010)). As a robustness check, the statistical validation and recovery analysis were additionally repeated on a filtered subsample excluding interactions with a margin below 0.10, to assess whether ambiguous assignments materially altered the reported effect sizes.

### 3.3 Feature importance and selection

Behavioral feature selection for cognitive state modeling followed a three-stage pipeline, designed to avoid arbitrary selection of candidate variables and to ensure that every included feature has prior statistical support before predictive modeling. A single-stage approach, such as training a Random Forest directly on all available variables, does not distinguish between features with genuine discriminative power and features whose importance emerges from spurious correlations or artifacts of the class distribution. The adopted strategy introduces a statistical filter prior to modeling that restricts the search space to variables with demonstrable effects across states, followed by two complementary fine-selection methods that validate one another to reduce the risk of selection driven by artifacts of any single procedure. The analysis was implemented in Python; the exact versions of the libraries used (scikit-learn, pandas, and numpy) are documented in the public code repository, and the random seed was fixed at 42 to ensure full reproducibility of the results.

In the first stage, a statistical preselection was applied to six candidate behavioral and historical variables: timeTaken, hintCount, consecutiveErrorsInRow, frPast5WrongCount, frPast8WrongCount, and totalFrTimeOnSkill. For each variable, the Kruskal–Wallis *H* statistic was computed across the six cognitive state groups, along with its effect size η^2^ (Tomczak and Tomczak, 2014). Only variables satisfying both *p* < 0.05 and η^2^>0.01 simultaneously were retained as candidate features. Five of the six candidates met both criteria; frPast5WrongCount was excluded at this stage, falling marginally below the effect-size threshold (η^2^ = 0.0099). No Bonferroni correction was applied at this stage, since the goal is an exploratory preselection filter rather than a confirmatory test: the conservative threshold of η^2^>0.01 serves to discard trivial effects without requiring adjustment for multiple comparisons.

In the second stage, the five statistically preselected variables were evaluated using a Random Forest classifier (Breiman, 2001), implemented in scikit-learn (Pedregosa et al., 2011). The model was configured with 120 trees, a maximum depth of 12 levels, and a minimum of 10 samples per leaf. The 120-tree configuration ensures stable importance estimates with negligible variance across runs (Breiman, 2001); the maximum depth of 12 and the minimum of 10 samples per leaf limit overfitting on a dataset with 799 student groups of heterogeneous size. Evaluation was performed using student-stratified cross-validation with *K* = 5 folds (Arlot and Celisse, 2010), ensuring that each student appeared exclusively in either the training or the test set to eliminate the risk of data leakage between interactions from the same student, a methodological limitation common in affect detection studies (Baker et al., 2012) (Equation 4):


$$\begin{array}{l} {\text{ACC}}_{CV}=\frac{1}{K}\overset{K}{\underset{k=1}{\sum }}\text{accuracy}\left({ŷ}_{k},y_{k}\right)\;\text{s.t.}G_{\text{train}}^{\left(k\right)}\cap G_{\text{test}}^{\left(k\right)}=\emptyset \end{array}$$ (4)

To establish a reference point, model performance was compared against a majority classifier (DummyClassifier, most_frequent) evaluated under the same cross-validation scheme, allowing the predictive contribution of the Random Forest over chance informed by the class distribution to be quantified.

In the third stage, fine selection of the optimal variable subset was performed on the five statistically preselected variables through the intersection of two complementary criteria. The first applies a Random Forest importance threshold of *I*_*j*_≥0.02, used as an exploratory heuristic to screen variables with very low relative importance, rather than as a theoretically derived noise threshold. The second applies RFECV (Guyon and Elisseeff, 2003), which iteratively evaluates cross-validated accuracy by removing one variable at each step and identifies the number of variables that maximizes predictive performance with maximum parsimony (Equation 5):


$$\begin{array}{l} F^{*}=\arg\underset{|F|\in \left\{2,\ldots ,p\right\}}{\max}{\text{ACC}}_{CV}\left(F\right) \end{array}$$ (5)

The internal RFECV estimator used reduced hyperparameters relative to the second-stage model: 60 trees, maximum depth 8, and a minimum of 20 samples per leaf. This simpler configuration is justified because RFECV requires training one model per variable subset at each cross-validation fold, so using the full model would multiply computation time without changing the relative ranking of variables, an objective for which simpler models are sufficient (Guyon and Elisseeff, 2003). All five variables exceeded the RF importance threshold, so no variable was excluded by this first criterion; RFECV, however, identified four variables as the optimal subset, excluding totalFrTimeOnSkill as redundant in the multivariate model despite its individual importance exceeding the threshold. The final subset F^*^ was determined by the intersection of both criteria, strengthening the validity of the resulting set by reducing the probability of including variables due to artifacts of a single selection procedure.

### 3.4 Circularity sensitivity analysis

The interaction-level variables used to construct the primary cognitive state are not fully independent of the variables analyzed as behavioral predictors, since timeTaken and hintCount are the same class of signal commonly used to train sensor-free affect detectors in this environment (Baker et al., 2004a, 2012). Their discriminative power in the present analysis could therefore partially reflect the detector's internal mapping rather than an independent behavioral pattern. As a sensitivity check, the feature selection and Random Forest classification procedure described above was repeated on the remaining three candidate variables (consecutiveErrorsInRow, frPast8WrongCount, totalFrTimeOnSkill), excluding timeTaken and hintCount entirely, and evaluated under the same student-stratified cross-validation scheme against the majority-class baseline.

### 3.5 Statistical validation

Differences in the distributions of the final subset F^*^ variables across the six cognitive states were evaluated using the Kruskal–Wallis test (Kruskal and Wallis, 1952), a nonparametric alternative to one-way ANOVA appropriate when distributions show pronounced skewness and normality cannot be assumed (Equation 6):


$$\begin{array}{l} H=\frac{12}{n\left(n+1\right)}\overset{k}{\underset{i=1}{\sum }}\frac{R_{i}^{2}}{n_{i}}-3\left(n+1\right) \end{array}$$ (6)

where *n* is the total number of observations, *k* the number of groups, *n*_*i*_ the size of group *i*, and *R*_*i*_ the sum of ranks for group *i*. Under the null hypothesis of identical distributions, *H* is approximately distributed as χ^2^ with *k*−1 degrees of freedom.

Since simultaneous comparisons were performed, one for each variable in the final subset F^*^, a Bonferroni correction (Dunn, 1961) was applied to control the family-wise Type I error rate. The adjusted threshold was calculated dynamically as a function of the number of variables selected by the pipeline (Equation 7):


$$\begin{array}{l} \alpha^{*}=\frac{\alpha }{\left|F^{*}\right|}=\frac{0.05}{\left|F^{*}\right|} \end{array}$$ (7)

This dynamic calculation ensures consistency between the number of comparisons performed and the applied correction threshold, regardless of how many variables result from the selection process. To identify which specific pairs of states differ significantly from one another, Dunn's *post-hoc* test (Dunn, 1961) with Bonferroni correction was applied to each variable that was significant in the global test, comparing all 62=15 possible pairs of cognitive states.

Statistical significance alone is insufficient to assess the practical relevance of a difference in large samples (Sullivan and Feinn, 2012), so effect size was quantified using the Kruskal–Wallis η^2^ following the procedure of Tomczak and Tomczak (2014) (Equation 8):


$$\begin{array}{l} \eta^{2}=\frac{H-k+1}{n-k} \end{array}$$ (8)

with the conventional interpretation thresholds: η^2^ < 0.01 (trivial), 0.01 ≤ η^2^ < 0.06 (small), 0.06 ≤ η^2^ < 0.14 (medium), and η^2^≥0.14 (large). The value of η^2^ was truncated at zero in cases where the expression produced negative values due to sampling variability.

### 3.6 Multilevel robustness check

The Kruskal–Wallis tests described above treat each interaction as an independent observation, which may inflate the resulting test statistics given the repeated-measures structure of the data. To assess whether this is the case, a mixed-effects linear model with a random intercept per student (Raudenbush and Bryk, 2002) was fitted for timeTaken and hintCount, the two variables with the largest effect sizes, using maximum likelihood estimation in statsmodels (Seabold et al., 2010) (Equation 9):


$$\begin{array}{r} y_{ij}=\beta_{0}+\overset{6}{\underset{c=2}{\sum }}\beta_{c}⊮\left[{\text{state}}_{ij}=c\right]+u_{j}+\varepsilon_{ij},\;u_{j}\sim N\left(0,\sigma_{u}^{2}\right),\\ \varepsilon_{ij}\sim N\left(0,\sigma_{\varepsilon }^{2}\right) \end{array}$$ (9)

where *y*_*ij*_ is the behavioral variable for interaction *i* of student *j*, and *u*_*j*_ is a student-level random intercept capturing between-student variability. To quantify the proportion of total variance attributable to between-student differences, the intraclass correlation coefficient was additionally computed for each model (Equation 10):


$$\begin{array}{l} \text{ICC}=\sigma_{u}^{2}/\left(\sigma_{u}^{2}+\sigma_{\varepsilon }^{2}\right) \end{array}$$ (10)

### 3.7 Markov transition modeling

The temporal dynamics of cognitive states were modeled using a first-order Markov chain (Rabiner, 1989). In this model, the probability of transitioning to the state at *t*+1 depends only on the state at *t*, a property that has been used in previous studies of affective dynamics in ITS (McQuiggan et al., 2010; Andres et al., 2019) and that is appropriate for capturing patterns of persistence and change between successive states in item-by-item interaction logs.

For each pair of consecutive states (*s*_*t*_, *s*_*t*+1_) within each student, observed transitions were tallied across the complete set of interactions. The transition frequency matrix was constructed by aggregating these observations at the population level (Equation 11):


$$\begin{array}{l} C_{ij}=\underset{g\in G}{\sum }\overset{T_{g}-1}{\underset{t=1}{\sum }}⊮\left[s_{t}^{\left(g\right)}=i,\;s_{t+1}^{\left(g\right)}=j\right] \end{array}$$ (11)

where *g* indexes students, *T*_*g*_ is the number of interactions of student *g*, and *i, j*∈*C*. Transition probabilities were estimated by normalizing each row of the frequency matrix (Equation 12):


$$\begin{array}{l} P_{ij}=\frac{C_{ij}}{\underset{j^{\prime }}{\sum }C_{ij^{\prime }}} \end{array}$$ (12)

The stationarity assumption of the Markov chain, under which transition probabilities remain constant throughout the session, was examined by comparing the transition matrices estimated for the initial third, the middle third, and the final third of each session. The maximum difference between self-transition probabilities across the three thirds was calculated as an indicator of non-stationarity. Differences below 0.10 were interpreted as indicating limited temporal variation.

### 3.8 Markov order comparison

The first-order specification assumes that transition probabilities depend only on the immediately preceding state. To assess whether this assumption adequately captures the temporal structure of the data, a second-order Markov model was additionally fitted, in which the transition probability to *s*_*t*+1_ was conditioned on both *s*_*t*−1_ and *s*_*t*_. Model fit was compared between the first- and second-order specifications using the Bayesian Information Criterion (BIC) (Schwarz, 1978) (Equation 13):


$$\begin{array}{l} \text{BIC}=-2\text{LL}+p\log\left(n\right) \end{array}$$ (13)

where LL is the model log-likelihood, *p* the number of free parameters [*k*(*k*−1) for the first-order model and *k*^2^(*k*−1) for the second-order model, with *k* = 6 states], and *n* the number of transition observations. Divergence between the two models was further characterized at the level of individual state-pair transitions using the total variation distance between the second-order conditional distribution and the corresponding first-order distribution, restricted to pairs with at least 200 observations.

### 3.9 Transition heterogeneity by student performance

The transition matrices reported above pool all 799 students into a single aggregate estimate, which could mask heterogeneity across learners of different performance levels. Comparing behavioral or sequential patterns between groups of students stratified by performance is an established approach in educational data mining, previously applied to differentiate learning behaviors between high- and low-performing students in other ITS environments (Kinnebrew et al., 2013). Following this precedent, students were stratified into performance terciles based on their mean correct-response rate across all recorded interactions, and a separate transition matrix was estimated for each tercile, applying the same frequency-counting and normalization procedure used for the aggregate matrix. Divergence between the lowest- and highest-performing terciles was quantified using the same total variation distance measure applied to the Markov order comparison, averaged across all rows of the transition matrix.

### 3.10 State duration and transition timing

The duration of each state episode was operationalized as the difference in seconds between the timestamp of the current item and that of the following item within the same student. This calculation was performed on consecutive pairs grouped by student, such that each student's last interaction does not produce a duration and is excluded from the analysis (Equation 14):


$$\begin{array}{l} d_{i}=t_{i+1}-t_{i}\;\text{(seconds)} \end{array}$$ (14)

The dataset contained records with null or negative time differences, produced by duplicate timestamps or by low server resolution when logging very rapid interactions. In these cases, a fallback procedure was applied that substitutes the time difference with the record's timeTaken value, a variable independently logged by the system as the time the student took to respond to the item (Equation 15):


$$\begin{array}{l} d_{i}=\{\begin{array}{cc} t_{i+1}-t_{i} & \text{if}t_{i+1}-t_{i}>0\\ {\text{timeTaken}}_{i} & \text{if}t_{i+1}-t_{i}\leq 0 \end{array} \end{array}$$ (15)

After applying the fallback, records with *d*_*i*_>7, 200 s were filtered out, considered inter-session pauses not representative of within-session cognitive dynamics. This 2-h threshold is consistent with standard practice in learning analytics for session delimitation (Kovanović et al., 2015). Records with *d*_*i*_ ≤ 0 after the fallback were likewise excluded. The resulting set of valid records was used for two complementary analyses: the distribution of durations by cognitive state, summarized through descriptive statistics (mean, median, and standard deviation), and the matrix of mean transition times between state pairs, calculated as the mean of *d*_*i*_ conditioned on the pair (*s*_*i*_, *s*_*i*+1_). The latter captures not only how long a student remains in a state, but also how long the transition from each origin state to each possible destination state takes.

### 3.11 Cognitive recovery analysis

Two central constructs were operationalized. A negative episode is any contiguous sequence of consecutive interactions by the same student in which the primary cognitive state belongs to the set of non-productive states *S*^−^ = {FRUSTRATED, GAMING, CONFUSED, BORED}. OFFTASK was excluded from this set given that its nature is behavioral rather than cognitive-affective (Baker et al., 2010). A recovery is the transition to the first subsequent item classified in the CONCENTRATING state, operationalized as the state of highest active cognitive engagement in the ITS literature (D'Mello and Graesser, 2011).

Before identifying episodes, each student's interactions were segmented into continuous learning sessions. Two consecutive interactions from the same student with a time gap exceeding 30 min were treated as belonging to distinct sessions. This criterion, consistent with the standard threshold used in educational data mining to delimit sessions (Kovanović et al., 2015), ensures that both the negative episode and the recovery occur within the same continuous session rather than across inter-session interruptions.

The recovery time for each episode starting at *t*_0_ and reaching CONCENTRATING at *t*_*r*_ was calculated as (Equation 16):


$$\begin{array}{l} \tau =t_{r}-t_{0}\;\text{s.t.}0<\tau \leq 1,800\text{s} \end{array}$$ (16)

The maximum window of 1,800 s corresponds to the same 30-min threshold used for session delimitation, ensuring consistency between both criteria. Recoveries outside this limit were discarded as they were considered transitions between distinct sessions rather than genuine cognitive recoveries.

For each episode of length *L* interactions, four behavioral predictors were extracted. Episode length *L* captures the depth of disengagement. Mean hint use $\bar{h}$ and mean consecutive error rate ē capture the student's active behavior during the episode (Baker et al., 2010). Mean detector confidence $\bar{c}$ reflects the system's certainty in the assignment of the negative state (Botelho et al., 2018) (Equation 17):


$$\begin{array}{r} \bar{h}=\frac{1}{L}\overset{L}{\underset{j=1}{\sum }}{\text{hintCount}}_{j},\\ ē=\frac{1}{L}\overset{L}{\underset{j=1}{\sum }}{\text{consecutiveErrorsInRow}}_{j},\bar{c}=\frac{1}{L}\overset{L}{\underset{j=1}{\sum }}{\text{conf}}_{j} \end{array}$$ (17)

Additionally, each episode was classified as pure if all of its interactions belonged to the same negative state, or mixed if it contained more than one distinct negative state. This distinction was retained as an episode-quality covariate, given that mixed episodes represent more complex disengagement trajectories whose recovery may be subject to different dynamics.

Episodes were classified as fast- or slow-recovering using the global median $\tilde{\tau }$ as a cutoff, a standard practice when the recovery time distribution is strongly skewed (Sullivan and Feinn, 2012). Differences between the two groups across the four behavioral predictors were evaluated using the Kruskal–Wallis test (Kruskal and Wallis, 1952), with Bonferroni correction for multiple comparisons (Dunn, 1961) and η^2^ as the effect size measure (Tomczak and Tomczak, 2014). Because recovery time in seconds is mechanically coupled to the per-item response pace of the originating state, episodes were additionally classified as fast or slow using the median split of episode length in number of interactions, providing a co-primary recovery outcome independent of clock-time pace.

## 4 Results

### 4.1 State distribution and prevalence

The CONCENTRATING state was the most frequent in the dataset, accounting for 345,526 interactions, equivalent to 57.85% of the total. The remaining 42.15% corresponded to states characterized by reduced or absent task engagement, distributed among GAMING (102,181 interactions; 17.11%), FRUSTRATED (68,292; 11.43%), OFFTASK (36,252; 6.07%), CONFUSED (36,068; 6.04%), and BORED (8,923; 1.49%). Taken together, GAMING and FRUSTRATED accounted for 28.54% of all recorded interactions, establishing themselves as the most frequent forms of disengagement in the analyzed context. Figure 2 presents the complete distribution of absolute frequencies and relative proportions for the six cognitive states.

**Figure 2:**
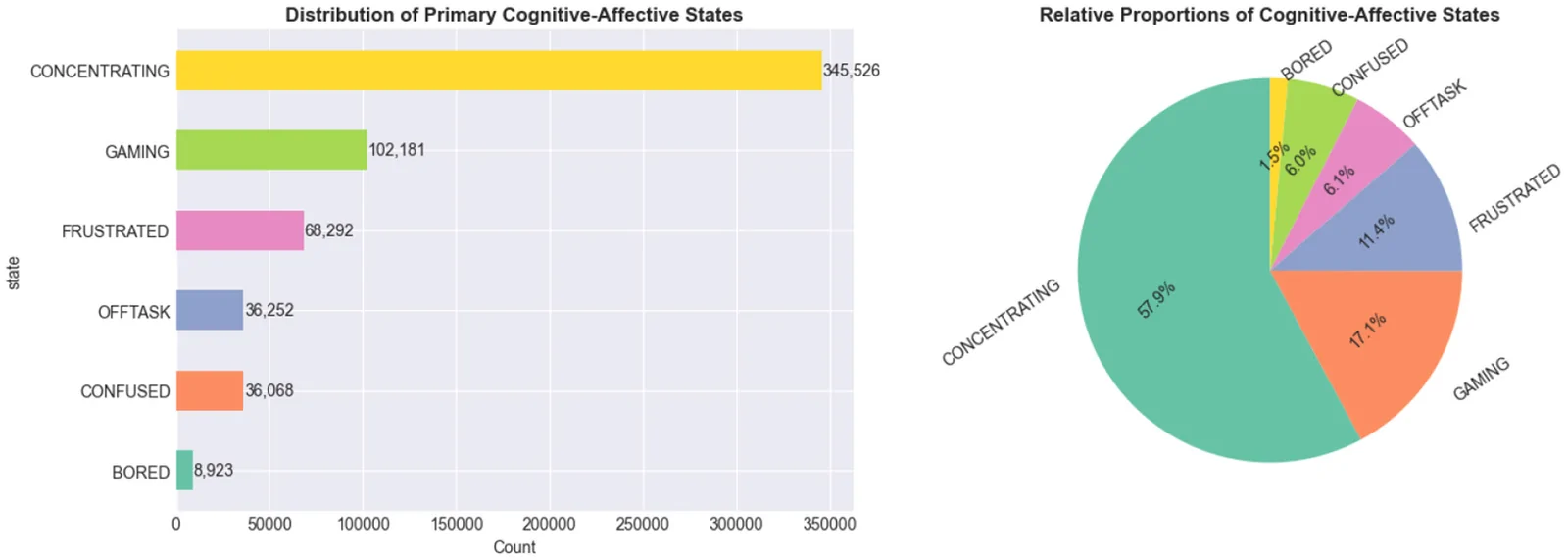
Distribution of cognitive-affective states across 597,242 interactions.

Analysis of the relative proportions reveals a clear hierarchical structure among the non-productive states. GAMING occurs at nearly twice the frequency of FRUSTRATED, which in turn is approximately twice as frequent as OFFTASK and CONFUSED, which present nearly identical proportions to one another (6.07% and 6.04%, respectively). This similarity between OFFTASK and CONFUSED contrasts with the distance separating them from BORED, whose 1.49% prevalence places it in a quantitatively distinct category relative to the other negative states. The gap between GAMING and BORED, the most and least frequent non-productive states, respectively, is 93,258 interactions, reflecting the internal heterogeneity of cognitive disengagement in terms of its incidence across the total record set.

The accumulation of 251,716 interactions in non-productive states underscores the extent of the disengagement phenomenon in the intelligent tutoring environment studied and establishes the empirical foundation for subsequent analyses. The GAMING state, with 102,181 records, alone exceeds the combined total of OFFTASK, CONFUSED, and BORED, while FRUSTRATED, with 68,292 interactions, constitutes the second-largest negative state in the sample. These record volumes ensure that the patterns identified within each category rest on sample sizes sufficient to support statistically robust inferences in the behavioral, temporal, and recovery analyses addressed in the following sections.

As a robustness check, the state distribution was recomputed using only interactions with an assignment margin of 0.10 or greater (480,551 of 597,242 interactions retained). The maximum absolute shift in prevalence was 4.98 percentage points, for CONCENTRATING (57.85% to 62.83%). In relative terms, however, the states were affected very unevenly: BORED lost 70.7% of its interactions under this filter, followed by FRUSTRATED (37.7%) and CONFUSED (33.7%), while CONCENTRATING (12.6%) and GAMING (23.0%) were comparatively stable. This indicates that BORED is disproportionately composed of low-confidence, ambiguous assignments relative to the other states, a pattern consistent with its heightened sensitivity observed in the transition-matrix and recovery-stratification robustness checks reported below.

### 4.2 Variable selection and feature importance

Of the six candidate variables, five simultaneously exceeded the significance and effect-size criteria in the statistical preselection; Past five wrong answers was the only one excluded, falling marginally below the effect-size threshold (η^2^ = 0.0099). The *H* statistics for the five retained variables ranged from *H* = 6, 608.82 (Total time on skill) to *H* = 141, 194.60 (Time taken), a wide range that already anticipates the importance hierarchy confirmed in the analyses that follow.

The associated effect sizes spanned from the small to the large range according to the conventional thresholds of Tomczak and Tomczak (2014): Time taken registered the largest effect (η^2^ = 0.236), followed by Hint count (η^2^ = 0.166), Consecutive errors (η^2^ = 0.058), Past eight wrong answers (η^2^ = 0.013), and Total time on skill (η^2^ = 0.011). This distribution establishes a clear hierarchy in the discriminative capacity of the set: response time and hint use emerge as the behavioral indicators with the greatest differentiating power across cognitive states, whereas the remaining variables, although statistically significant, operate in the small-effect range with a substantially smaller differentiating contribution.

The Random Forest classifier trained on the five statistically preselected variables achieved a mean accuracy of 0.667 ± 0.002 under student-stratified cross-validation with *K* = 5 folds, compared to 0.579 ± 0.007 for the majority classifier evaluated under the same scheme, representing an absolute improvement of 0.089 and a relative improvement of 15.4% over the baseline. The model's importance profile, presented in Figure 3, reveals a pronounced hierarchical structure, in which Time taken dominated the ranking with an importance of 0.584, nearly tripling that of the second most relevant variable, Hint count (0.226); together, both variables account for 81.0% of the model's total importance. The remaining three variables were distributed across a narrow range between 0.053 and 0.080: Consecutive errors (0.080), Past eight wrong answers (0.057), and Total time on skill (0.053), indicating that their individual contribution to discriminative power is comparatively marginal. This distribution suggests that a student's cognitive state manifests primarily in response pace and help-seeking behavior, while accumulated error history and time-on-skill operate as lower-resolution background signals.

**Figure 3:**
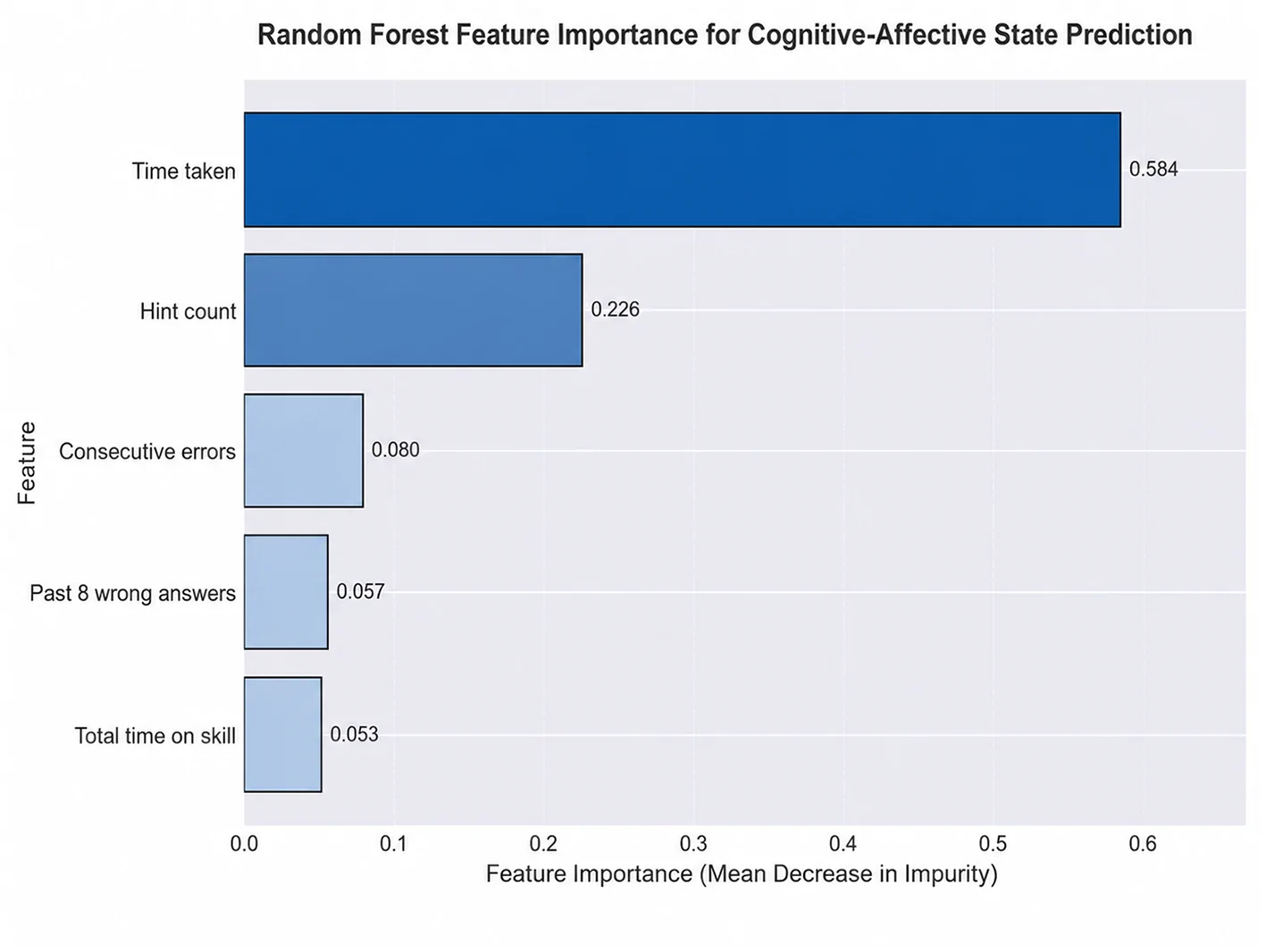
Feature importance from the Random Forest classifier for predicting the primary cognitive-affective state (five statistically preselected variables).

Figure 4 shows how the five preselected variables were narrowed down to a final subset, applying two selection criteria independently and then comparing them. The left panel shows the RF importance profile with the cutoff line at *I* = 0.020: all five variables exceeded this threshold and were retained by the importance criterion alone. Bar color in this panel reflects inclusion in the final intersected set rather than the importance criterion in isolation: four bars appear in green, corresponding to variables retained after both criteria are combined, while Total time on skill appears in red, indicating its exclusion by RFECV despite individually exceeding the RF importance threshold. The right panel presents the RFECV accuracy curve as a function of the number of included variables, identifying four variables as the optimal subset that maximizes cross-validated accuracy with maximum parsimony, the point marked by the vertical red dashed line.

**Figure 4:**
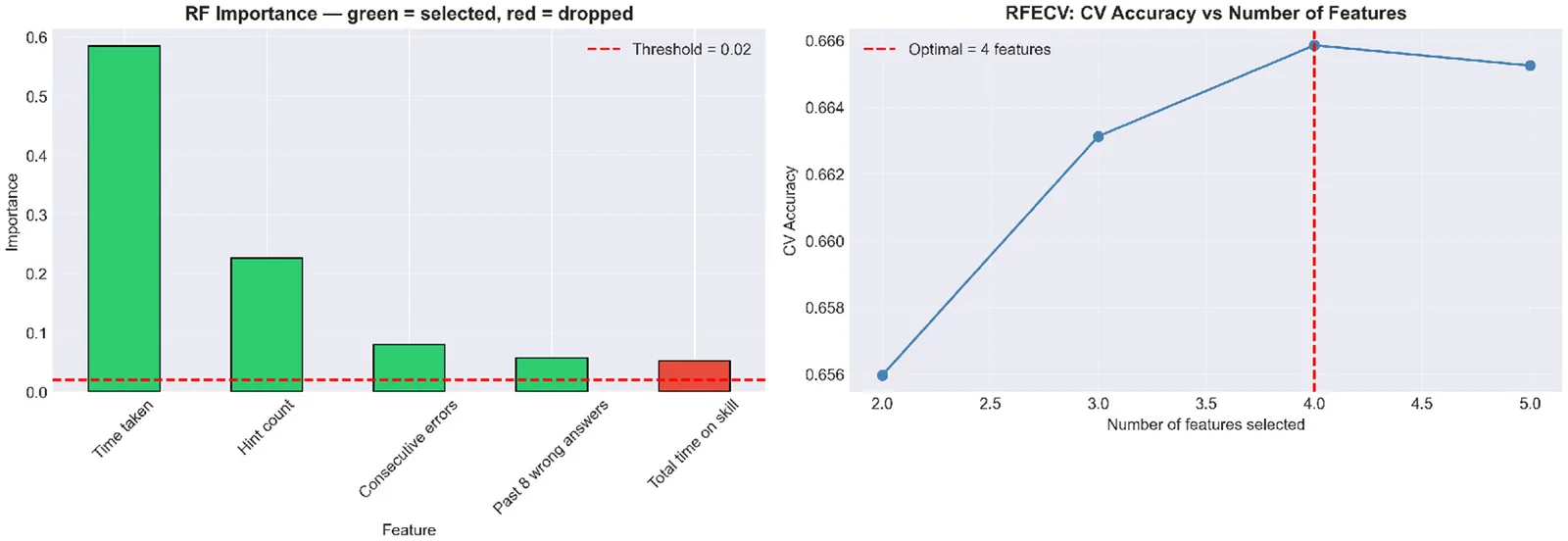
RF importance profile with selection threshold, colored by inclusion in the final intersected set **(left)**, and RFECV accuracy curve as a function of the number of selected variables **(right)**.

The final set F^*^ was determined by the intersection of both criteria: Time taken, Hint count, Consecutive errors, and Past eight wrong answers. The exclusion of Total time on skill is methodologically coherent: although it individually exceeded the RF importance threshold, RFECV identified it as redundant in the multivariate model, its contribution being largely absorbed by the combination of the remaining four variables once evaluated jointly rather than in isolation. These four variables constitute the input for all behavioral, temporal, and cognitive recovery analyses reported in the following sections. The predictors avg_hints and avg_errors were computed conditionally: avg_hints was included only if hintCount was part of the final subset F^*^, and avg_errors only if consecutiveErrorsInRow was also part of it, avoiding the introduction of statistically unvalidated variables into the recovery analysis at this stage. Both base variables were retained in F^*^, so both predictors were included.

Table 3 reports the results of the circularity sensitivity check: removing timeTaken and hintCount reduced accuracy from 0.667 to 0.583, a drop of 0.084 (12.6% relative), leaving the reduced model only 0.004 above the majority-class baseline. This gap is informative on two counts. First, it establishes that these two variables carry the substantial majority of the model's discriminative power, rather than merely ranking highest among several comparably informative predictors; the three remaining variables, evaluated jointly, approach the discriminative capacity of guessing the modal class. Second, within the reduced model itself, totalFrTimeOnSkill (importance = 0.464) and consecutiveErrorsInRow (0.409) absorbed most of the residual signal, while frPast8WrongCount contributed comparatively little (0.127), reproducing at a smaller scale the same concentration-of-importance pattern observed in the full model.

**Table 3:** Random Forest accuracy under the circularity sensitivity check.

Model	Variables included	Accuracy (CV)
Full model	timeTaken, hintCount, consecutiveErrorsInRow, frPast8WrongCount, totalFrTimeOnSkil	0.667 ± 0.002
No-circularity model	consecutiveErrorsInRow, frPast8WrongCount, totalFrTimeOnSkill	0.583 ± 0.006
Majority-class baseline	—	0.579 ± 0.007

### 4.3 Statistical validation

Statistical validation confirmed that Time taken, Hint count, Consecutive errors, and Past eight wrong answers (the four variables in the final set F^*^) present significant differences across the six cognitive states after applying the Bonferroni correction. With four simultaneous comparisons, the adjusted threshold was established at α^*^ = 0.050/4 = 0.0125. All four variables had *p* < 0.001, remaining below the adjusted significance threshold; therefore, none were discarded at this confirmatory stage. The left panel of Figure 5 shows the Kruskal–Wallis *H* statistics by variable: Time taken registered the highest value (*H* = 141, 194.60), followed by Hint count (*H* = 99, 270.55), Consecutive errors (*H* = 34, 355.76), and Past eight wrong answers (*H* = 7, 694.96). This hierarchy is consistent with the Random Forest importances, reinforcing the coherence between both approaches.

**Figure 5:**
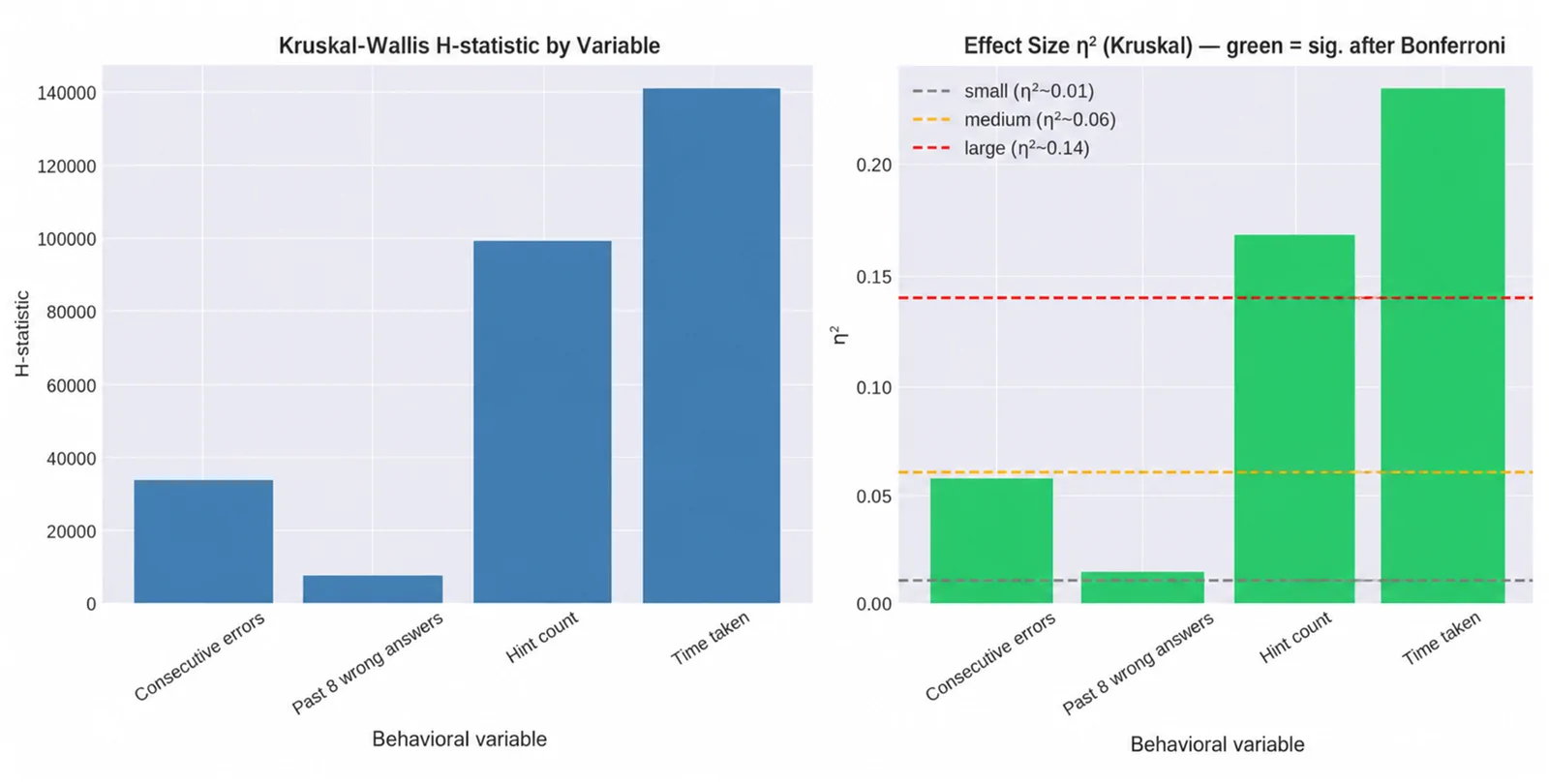
Kruskal–Wallis *H*-statistic and effect size η^2^ for the final feature set across cognitive-affective states.

The right panel of Figure 5 presents the Kruskal–Wallis η^2^ effect sizes for each variable, with reference lines for small (η^2^ = 0.010), medium (η^2^ = 0.060), and large (η^2^ = 0.140) effects according to Tomczak and Tomczak (2014); all bars appear in green, confirming significance after the Bonferroni correction. Time taken reached a large effect (η^2^ = 0.236), the only variable to substantially exceed the large-effect threshold, indicating that response time distributions across cognitive states differ substantially and are practically interpretable. Hint count registered an effect likewise classified as large (η^2^ = 0.166), above the η^2^ = 0.140 reference line. The two remaining variables operated in the small-effect range: Consecutive errors (η^2^ = 0.058), just below the medium-effect boundary, and Past eight wrong answers (η^2^ = 0.013), with values exceeding the minimum inclusion threshold but without reaching medium-magnitude practical relevance.

Dunn's *post-hoc* test with Bonferroni correction, applied to Time taken as the variable with the largest effect size, revealed significant differences across all 15 possible pairs of cognitive states (*p* < 0.001 in all cases after adjustment). This result indicates that response time robustly distinguishes each state from all others, with no pair of states sharing an equivalent temporal distribution. The universal significance across all 15 pairs establishes that the six cognitive states occupy differentiated positions in the space of student temporal behavior, a result that validates the operationalization of the primary state via the argmax rule and underpins the use of these variables as predictors in the temporal dynamics and cognitive recovery analyses presented next.

Table 4 compares Kruskal–Wallis effect sizes for the final feature set under the full dataset and under a subsample excluding the 19.5% of interactions with an assignment margin below 0.10 (480,551 of 597,242 records retained). Time taken and Hint count, the two variables carrying the largest share of discriminative power in the main analysis, showed the smallest shifts in either direction (Δη^2^ = +0.005 and −0.007, respectively) and remained classified as large effects. Past eight wrong answers was essentially unchanged (Δη^2^ < 0.001). Consecutive errors was the only variable to shift by a magnitude worth noting, moving from η^2^ = 0.058 to 0.089 and crossing into the medium-effect range; its *H* statistic increased from 34,355.76 to 42,549.41 despite the smaller sample, suggesting that low-margin assignments were disproportionately associated with weaker, noisier values of this variable rather than simply diluting an otherwise stable signal. Overall, the effect sizes remained largely stable after excluding ambiguous assignments. Hint count showed a small decrease from 0.166 to 0.159 but remained in the large-effect category, which is the opposite of what would be expected if detector uncertainty were inflating the reported effects.

**Table 4:** Kruskal–Wallis effect sizes with and without low-margin assignments.

Variable	η^2^ (all data)	η^2^ (margin ≥0.10)	*Δη* ^2^
Time taken	0.236	0.241	+0.005
Hint count	0.166	0.159	−0.007
Consecutive errors	0.058	0.089	+0.031
Past eight wrong answers	0.013	0.013	< 0.001

Table 5 reports the mixed-effects coefficients for Time taken and Hint count, the two variables with the largest effect sizes in the original Kruskal–Wallis analysis. Every state-level coefficient remained significant at *p* < 0.001 after accounting for the random intercept, and the ordering of states was preserved: GAMING showed the largest negative deviation from BORED in Time taken (−44.966 s) and OFFTASK the largest positive one (+61.866 s), consistent with the response-time hierarchy reported above. For Hint count, GAMING was the only negative state associated with an *increase* relative to BORED (+0.685), while the remaining four states showed decreases of comparable magnitude (−0.452 to −1.086). The intraclass correlation coefficients were modest for both variables (ICC = 0.052 for Time taken, 0.056 for Hint count), indicating that between-student differences account for only about 5% of total variance in either variable, with the remaining variance occurring within students across interactions.

**Table 5:** Mixed-effects model coefficients (reference category: BORED) and intraclass correlation.

State (vs. BORED)	Time taken, coef. (s)	Hint count, coef.
Intercept (BORED)	54.666	1.759
CONCENTRATING	−32.418	−0.621
CONFUSED	−31.367	−0.841
FRUSTRATED	−27.204	−1.086
GAMING	−44.966	+0.685
OFFTASK	+61.866	−0.452
ICC	0.052	0.056

### 4.4 Temporal dynamics and learning impact

The first-order Markov transition matrix, presented in Figure 6, reveals a markedly heterogeneous persistence structure across the six cognitive states. Self-transition probabilities, located along the main diagonal, ranged from 0.245 for BORED to 0.816 for GAMING, a difference reflecting qualitatively distinct profiles of temporal stability. GAMING registered the highest self-transition probability in the set, indicating that more than eight out of every 10 interactions in that state are followed by another interaction in the same state. CONCENTRATING was the second most stable state, with a self-transition probability of 0.784, followed at a distance by CONFUSED (0.481), FRUSTRATED (0.444), and OFFTASK (0.352).

**Figure 6:**
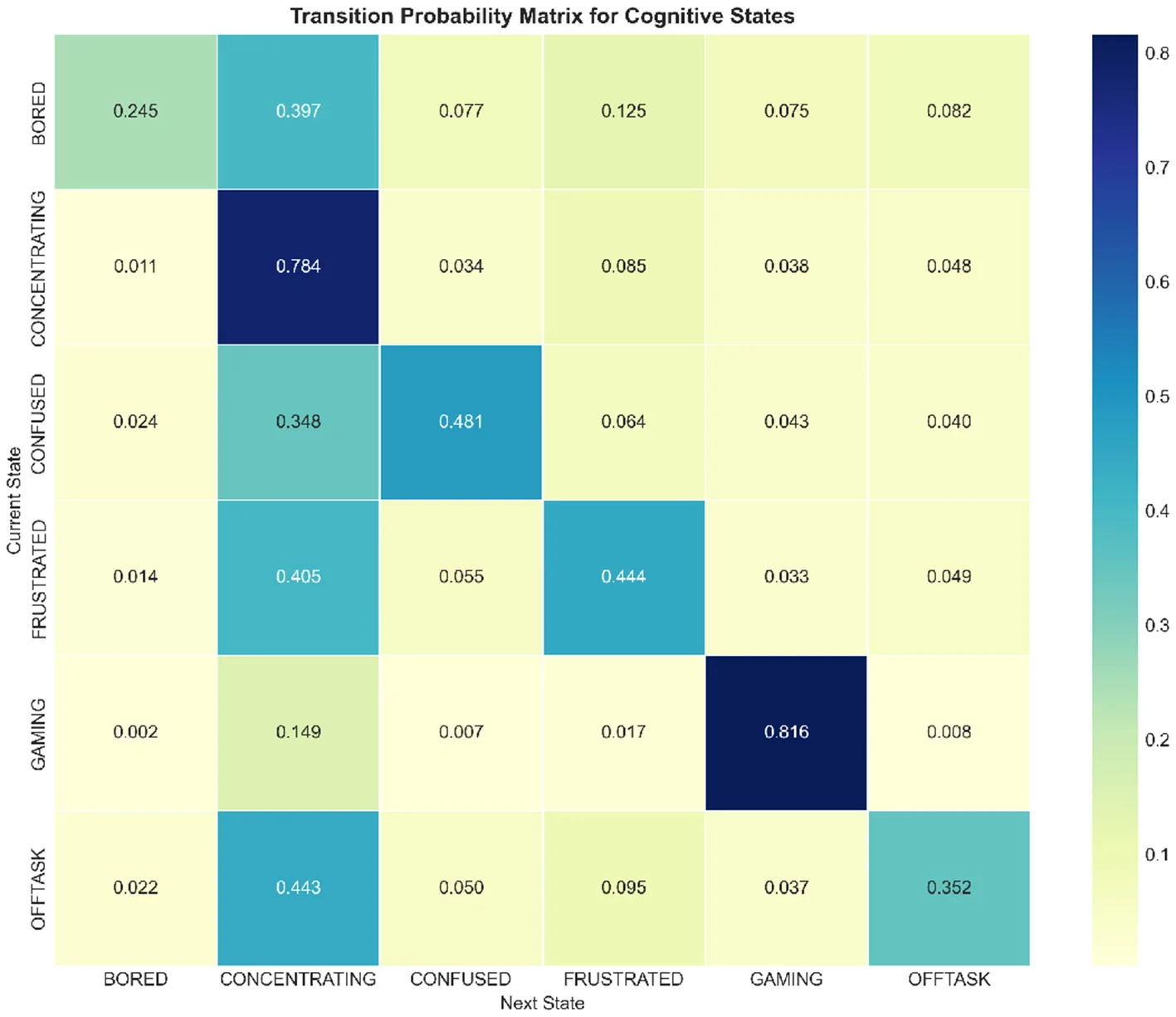
Transition probability matrix for cognitive-affective states estimated from 597,242 interactions.

BORED constituted the structural exception in the set: with a self-transition probability of only 0.245, it is the only state for which remaining in place is not the most probable transition, being instead replaced by a transition to CONCENTRATING (0.397) as the majority destination, followed by transitions to FRUSTRATED (0.125), to GAMING (0.075), to OFFTASK (0.082), and to CONFUSED (0.077). This pattern of high transitory dispersion suggests that boredom episodes are inherently unstable and tend to resolve spontaneously, though not necessarily toward the productive state. Temporal variation was assessed by comparing the matrices for the three session thirds: the maximum difference in self-transition probabilities across thirds was 0.045, corresponding to OFFTASK, a value below 0.100, suggesting limited temporal variation without validating the first-order Markov assumption.

Analysis of the off-diagonal transitions reveals that CONCENTRATING operates as the preferred destination from all negative states, albeit with markedly different intensities. From OFFTASK, the probability of transitioning to CONCENTRATING (0.443) exceeds the state's own self-transition probability (0.352), making it the most frequent exit from that state and suggesting that behavioral disconnection is frequently transitory. From FRUSTRATED, the probability of transitioning to CONCENTRATING was 0.405, close to its self-transition probability (0.444), indicating that nearly four out of every 10 frustration episodes are followed by recovery to the productive state on the immediately subsequent item. From CONFUSED, the transition to CONCENTRATING (0.348) was the second most probable after self-transition (0.481), while the transition to FRUSTRATED (0.064) was notably low, suggesting that confusion does not systematically escalate into frustration.

Figure 7 presents the differential impact of cognitive states on learning performance. The left panel shows the correct-response rate by state: CONCENTRATING registered the highest rate (46.0%), followed by FRUSTRATED (35.0%), OFFTASK (22.3%), GAMING (14.8%), CONFUSED (13.3%), and BORED (5.7%). The 11.0-percentage-point gap between CONCENTRATING and FRUSTRATED indicates that even the best-performing negative state operates substantially below the productive state. Notably, GAMING and CONFUSED present nearly equivalent rates, differing by only 1.5 percentage points, despite representing qualitatively distinct forms of disengagement: GAMING entails rapid responding without content processing, whereas CONFUSED reflects active cognitive difficulty with the material. BORED registered the lowest rate in the set (5.7%), barely a tenth of the CONCENTRATING rate, reinforcing its characterization as the state of lowest functional cognitive engagement with the task.

**Figure 7:**
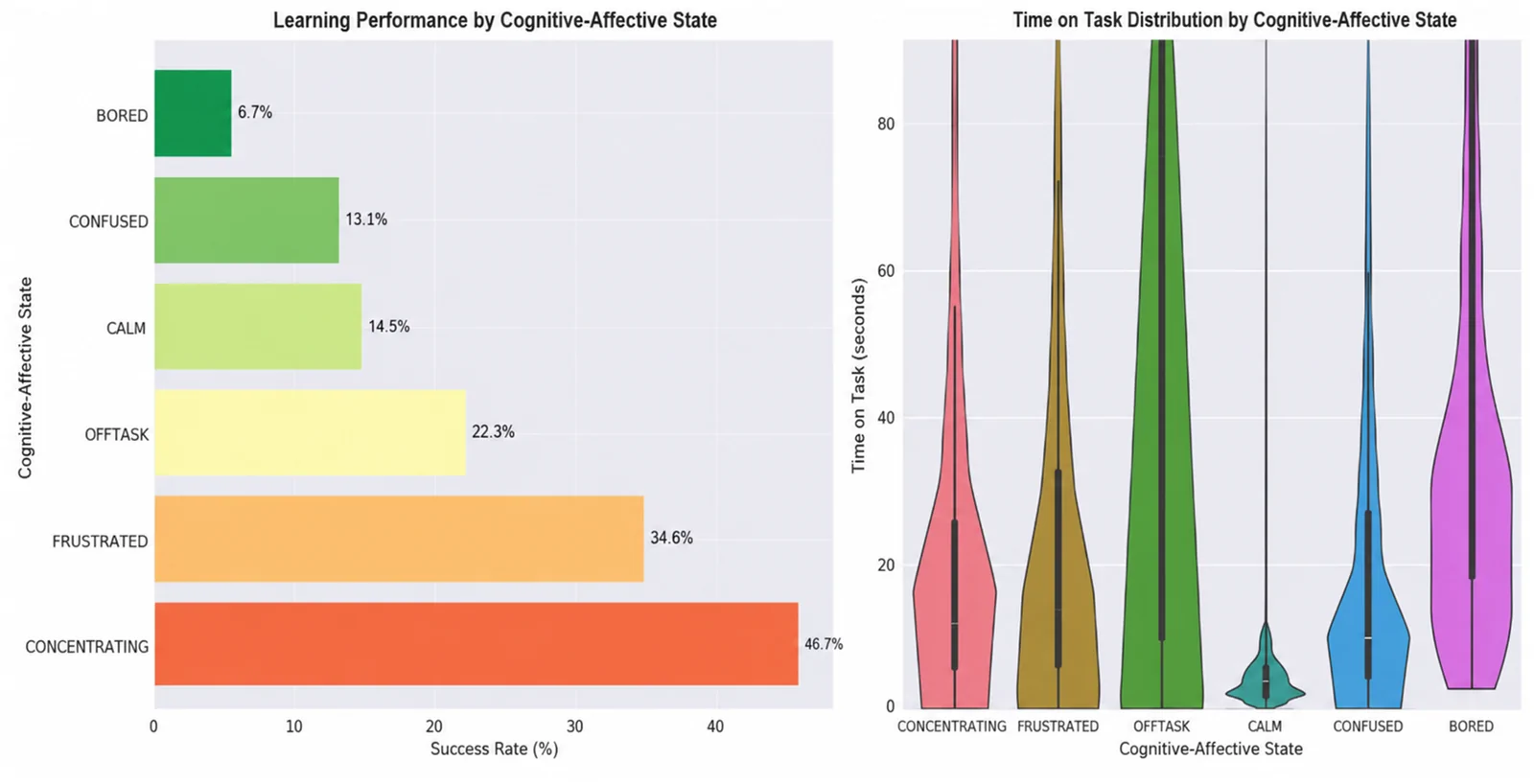
Learning performance and time-on-task distribution by cognitive-affective state.

The right panel presents response-time distributions by state using violin plots truncated at the 95th percentile. OFFTASK exhibited the broadest and most asymmetric distribution, with an upper tail extending to approximately 90 s, reflecting prolonged disconnection episodes. FRUSTRATED and BORED showed distributions of intermediate width with elongated bodies toward medium-high values, consistent with greater processing time in the face of difficulty. CONCENTRATING presented a compact distribution with a visual median close to 10 s, reflecting fluid and efficient content processing. GAMING exhibited the narrowest and most compressed violin in the set, with mass concentrated at the lowest values on the axis and virtually no density above 10 s, confirming the pattern of rushed responding without temporal investment in the item that structurally characterizes this state. CONFUSED presented an equally narrow and compressed distribution, a counterintuitive result given that cognitive difficulty would be expected to associate with longer response times, suggesting that students in this state respond quickly and incorrectly without investing additional time in processing the item.

Table 6 compares the two specifications. Despite the six-fold increase in free parameters, the second-order model achieved a lower BIC by 19,880 points, indicating that the transition probability to *s*_*t*+1_ is not fully determined by *s*_*t*_ alone. This gap is large enough that it is unlikely to be an artifact of sample size: with 597,242 observations, the BIC complexity penalty (*p*log*n*≈13.3*p*) is modest relative to the log-likelihood improvement, so the preference for the second-order model reflects genuine additional structure rather than overfitting. Total variation distance between the first- and second-order conditional distributions, computed for state pairs with at least 200 observations, was highest for transitions into and out of BORED and CONFUSED (TVD = 0.226 for CONFUSED → BORED and 0.195 for BORED → CONFUSED) and for the high-frequency pair CONCENTRATING → GAMING (TVD = 0.177, *n* = 12, 972). In each of these cases, conditioning on the state two steps prior shifted the probability of returning to CONCENTRATING downward and increased the probability of remaining within or re-entering a negative state, relative to the first-order estimate.

**Table 6:** Model fit comparison between first- and second-order Markov specifications.

Model	Log-likelihood	Free parameters	BIC
First-order	−538, 683.5	30	1, 077, 765.9
Second-order	−527, 746.2	180	1, 057, 885.9

As a further robustness check, the first-order transition matrix was recomputed using only interactions with an assignment margin of 0.10 or greater (480,551 of 597,242 interactions retained). The core structural pattern was preserved: GAMING remained the most persistent state (self-transition probability 0.817 vs. 0.816 in the full sample) and CONCENTRATING remained highly stable (0.822 vs. 0.784). BORED showed the largest sensitivity to this filtering (maximum absolute difference of 0.081 across its row), with its self-transition probability rising from 0.245 to 0.326 and its exit rate to CONCENTRATING falling correspondingly.

This instability is not isolated to the transition matrix: BORED's prevalence was also the most sensitive of all six states to the same margin filter, losing 70.7% of its interactions when restricted to confidently assigned cases. This suggests that BORED, as operationalized by this detector, is disproportionately drawn from the least confidently distinguishable region of the underlying probability space, a pattern discussed further below.

Table 7 compares key transition probabilities across the three performance terciles (267, 266, and 266 students, respectively; mean accuracy 0.274, 0.350, and 0.490). Both negative-state self-transitions decreased monotonically with student performance, from 0.827 to 0.787 for GAMING and from 0.271 to 0.217 for BORED, while the corresponding recovery probabilities to CONCENTRATING increased monotonically over the same range, from 0.140 to 0.177 and from 0.368 to 0.434, respectively. Self-transition into CONCENTRATING, by contrast, remained essentially constant across terciles (range = 0.010), indicating that the productive state's stability does not vary with student performance even though the stickiness of negative states does. The average total variation distance between the Low and High terciles across the full transition matrix was 0.104, smaller than the largest order-2 divergences reported above but well above zero, confirming genuine heterogeneity. Critically, GAMING remained the most persistent state in every tercile (0.787–0.827), indicating that its structural resistance to spontaneous recovery is not an artifact of aggregating across a heterogeneous student population.

**Table 7:** Key transition probabilities by student performance tercile.

From	To	Low	Mid	High	Range
GAMING	GAMING	0.827	0.805	0.787	0.040
BORED	BORED	0.271	0.243	0.217	0.054
BORED	CONCENTRATING	0.368	0.395	0.434	0.066
GAMING	CONCENTRATING	0.140	0.157	0.177	0.037
CONCENTRATING	CONCENTRATING	0.782	0.781	0.791	0.010

### 4.5 State duration and transition timing

The state duration analysis was performed on 585,607 valid records out of the 597,242 total, after applying the timeTaken fallback procedure to 5,465 rows with null or negative time differences and excluding records with durations exceeding 7,200 s; this exclusion affected only 1.8% of the 596,443 eligible consecutive pairs. This same filtered set of valid records is used for both the duration analysis presented next and the transition-time matrix in Figure 8. Figure 9 presents duration distributions by state using violin plots truncated at the 95th percentile. OFFTASK registered the highest mean duration in the set (110.26 s; median = 67.0; SD = 141.79), with a wide-bodied violin in the 20–100 s range and an upper tail extending to approximately 110 s, reflecting behavioral disconnection episodes of highly variable duration across interactions.

**Figure 8:**
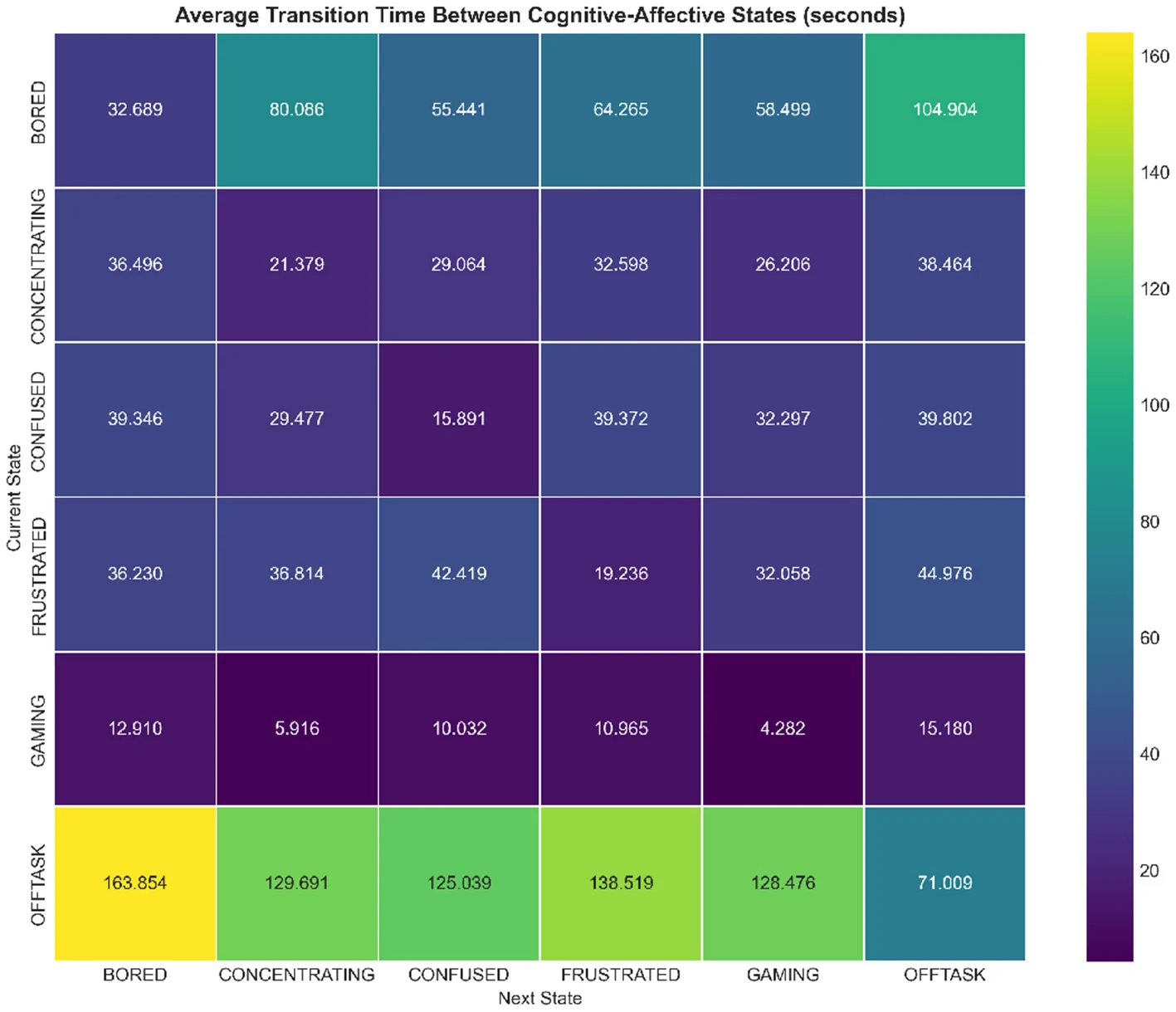
Average transition time between cognitive-affective states (seconds).

**Figure 9:**
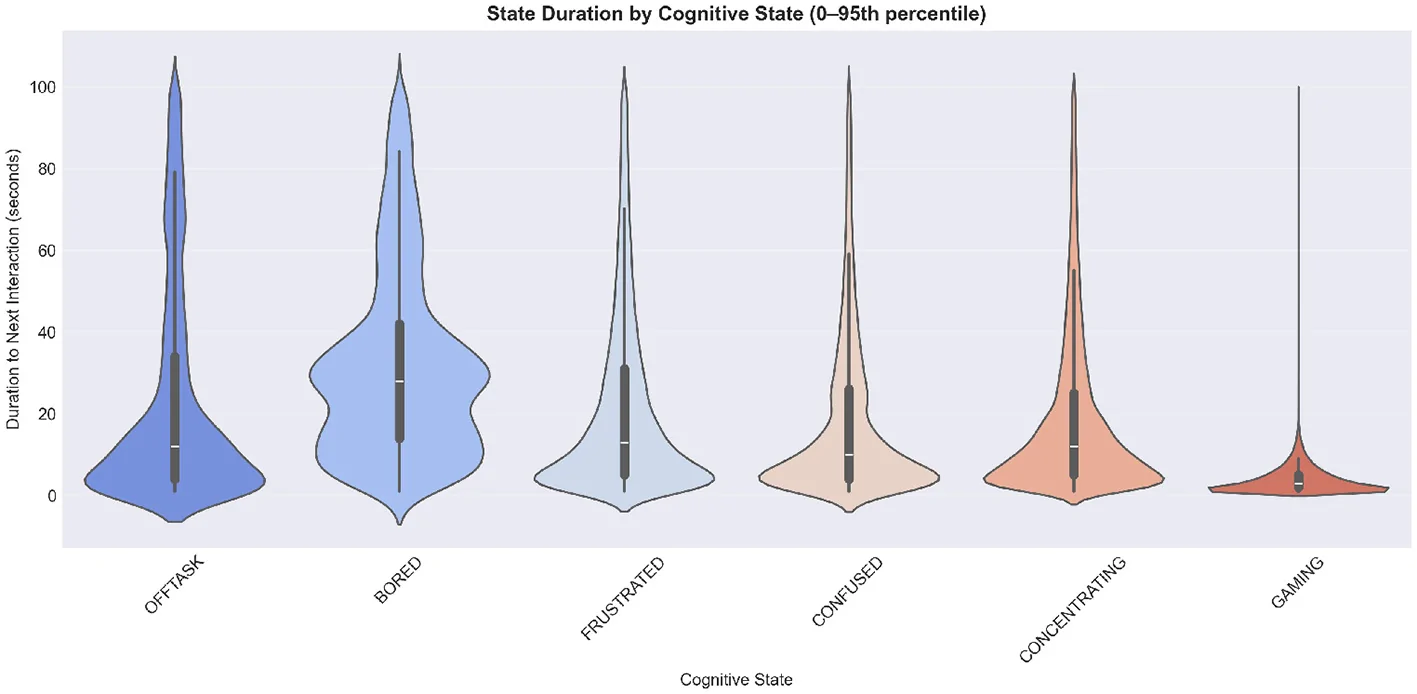
State duration distributions by cognitive-affective state (0–95th percentile).

BORED was the state with the second-highest mean duration (64.69 s; median = 37.0; SD = 144.15), with a standard deviation exceeding even its mean, indicating an extremely asymmetric distribution. The BORED violin presents a wide body in the 10–75 s range with a pronounced bulge toward medium values, followed by a thin upper tail, reflecting that most boredom episodes have intermediate duration, but with occasionally very prolonged cases. The gap between the mean and median for BORED is the second highest in the set after OFFTASK, confirming the positive skewness of its distribution.

The active cognitive-difficulty states FRUSTRATED and CONFUSED presented similar mean durations to one another (29.52 and 24.29 s, respectively), with low medians of 14.0 and 10.0 s. The violins for both states show elongated, narrow bodies concentrating probability mass at low values, with upper tails reaching 107 s but with very little density in that range. This similarity in central tendency between FRUSTRATED and CONFUSED contrasts with their distinct behavioral profiles, suggesting that item duration alone is insufficient to differentiate between these two forms of cognitive difficulty.

CONCENTRATING showed a mean duration of 23.72 s (median = 12.0; SD = 69.03), structurally comparable to CONFUSED and FRUSTRATED in terms of central tendency. The proximity of the mean duration between CONCENTRATING and CONFUSED indicates that time invested per item does not discriminate between the productive state and states of difficulty, reinforcing the relevance of behavioral variables such as hint count and consecutive errors as more precise discriminators of cognitive state. GAMING registered the shortest duration in the set by a wide margin (4.78 s; median = 3.0; SD = 23.96), with an extremely compressed violin at the lowest values on the axis and virtually no probability mass above 10 s, consistent with the rushed-response profile.

Figure 8 presents the matrix of mean transition times between state pairs, in seconds. The row corresponding to OFFTASK concentrates the highest values in the entire matrix, with times ranging from 71.0 s for remaining in the same state to 163.9 s for the transition to BORED. Transition times from OFFTASK to the dataset's most frequent states are similarly elevated: 129.7 s to CONCENTRATING, 138.5 s to FRUSTRATED, 128.5 s to GAMING, and 125.0 s to CONFUSED. This uniformly high exit latency from OFFTASK, regardless of the destination state, indicates that behavioral disconnection episodes are characterized by prolonged inter-item pauses that precede any transition.

At the opposite extreme, the GAMING row concentrates the lowest values in the entire matrix, with times ranging from 4.3 s for remaining in the same state to 15.2 s for the transition to OFFTASK. The self-transition value for GAMING is the absolute minimum in the entire matrix, and transition times from that state to CONCENTRATING (5.9 s), to CONFUSED (10.0 s), and to FRUSTRATED (11.0 s) are similarly reduced. The CONCENTRATING, CONFUSED, and FRUSTRATED rows showed moderate and relatively homogeneous times across destinations, with minimum values for self-transition: 21.4 s for CONCENTRATING, 15.9 s for CONFUSED, and 19.2 s for FRUSTRATED, confirming that remaining in the same state is temporally more efficient than any transition to a different state. The BORED row presented elevated times toward all destinations, especially toward OFFTASK (104.9 s) and toward CONCENTRATING (80.1 s), while its self-transition time was the lowest in its row (32.7 s), consistent with the pattern of high transitory instability identified in the Markov matrix.

### 4.6 Cognitive state recovery analysis

The cognitive recovery analysis identified 63,646 disengagement episodes distributed across 797 students; the remaining two presented no negative episodes over the observation period. The 597,242 interactions were segmented into 7,957 continuous learning sessions, with a mean of 10 sessions per student (median = 10; maximum = 36). Of the identified episodes, 52,151 were classified as pure (81.9%), that is, composed exclusively of interactions in a single negative state, while 11,495 (18.1%) were classified as mixed, containing more than one distinct negative state. Among mixed episodes, the most frequent combination was CONFUSED and FRUSTRATED (3,793 episodes), followed by FRUSTRATED and GAMING (2,756), CONFUSED and GAMING (1,349), and BORED and FRUSTRATED (1,071), suggesting that disengagement co-occurrences follow structured, non-random patterns.

The left panel of Figure 10 presents the distribution of recovery time to CONCENTRATING by originating negative state, through boxplots evidencing substantial differences across states. GAMING registered the fastest recovery in the set, with a mean of 37 s and a median of 18 s (SD = 69), and a boxplot compressed toward the lowest values with numerous outliers in the upper range. CONFUSED presented intermediate recovery times (mean = 70.5 s; median = 38.0; SD = 105.4), with a wider boxplot than GAMING but substantially more compact than the remaining states. FRUSTRATED registered a mean of 78 s (median = 42.0; SD = 117.7), with a distribution profile similar to CONFUSED, though with a slightly wider box and a higher density of outliers in the upper range. BORED presented the longest recovery times in the set (mean = 131.8 s; median = 108.0; SD = 142.1), with a markedly elevated boxplot relative to the other states and a box extending primarily across the 50–250 s range, indicating that boredom episodes require considerably more time to resolve toward the productive state than any other form of disengagement.

**Figure 10:**
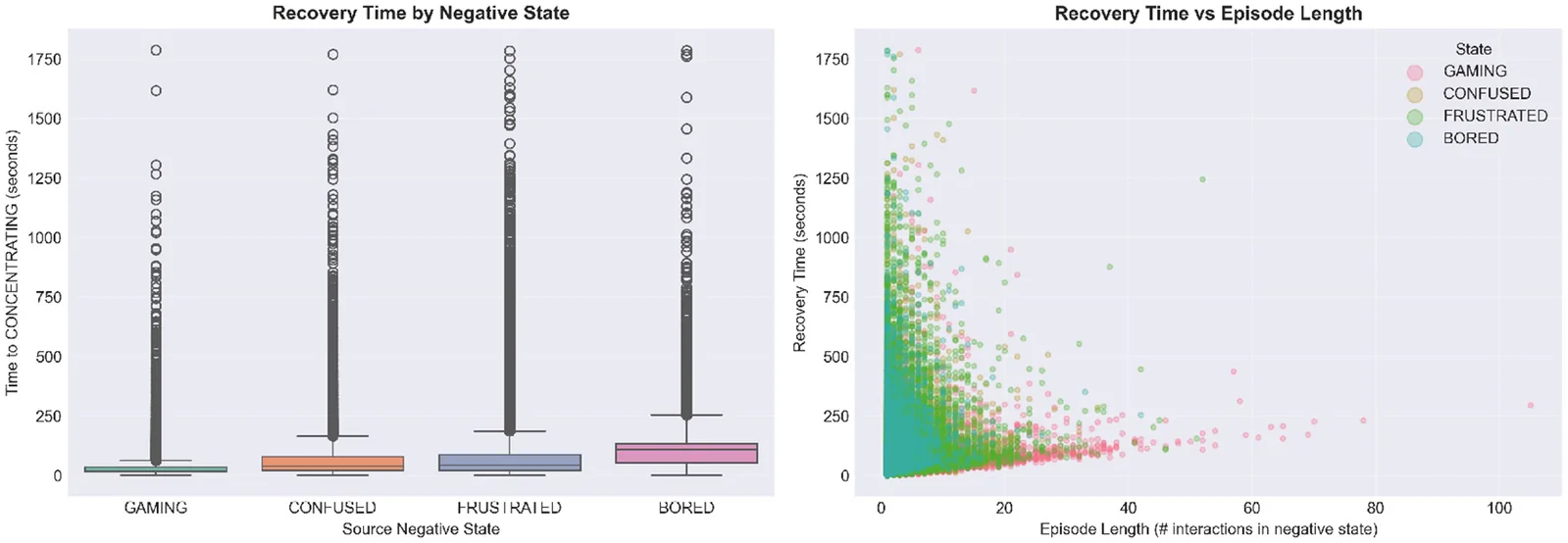
Recovery time to CONCENTRATING by negative source state and episode length.

The right panel of Figure 10 presents the relationship between recovery time and episode length in number of interactions, colored by originating negative state. The point cloud reveals a pronounced decreasing relationship in the 1–15 interaction range: the shortest episodes present the greatest variability in recovery times, with values extending from near zero to 1,800 s, while longer episodes converge toward lower and more homogeneous recovery times. Points corresponding to GAMING (pink) concentrate in the lower portion of the vertical axis across the entire range of lengths, confirming that GAMING episodes recover consistently faster regardless of duration. FRUSTRATED (green) and CONFUSED (yellow) episodes dominate the cloud in the intermediate-length range, with greater vertical dispersion. BORED (teal) episodes appear sparsely represented at lengths above 20 interactions, consistent with the inherently unstable character of that state identified in the transition analysis.

The global median recovery time, used as the cutoff threshold to classify fast and slow recoveries, was 36 s. Of the 63,646 total episodes, 31,695 were classified as fast recovery and 31,951 as slow recovery, with a nearly symmetric distribution around the threshold. Figure 11 presents the comparison of the four predictors between the two groups using mean values. Episode length was greater for slow recoveries (mean = 3.77 interactions) than for fast recoveries (mean = 2.74), indicating that episodes of greater disengagement depth tend to be associated with longer recovery times. Mean hint use showed the inverse pattern: fast recoveries presented greater hint use (mean = 1.508) than slow recoveries (mean = 0.947), suggesting that active help-seeking during the episode facilitates the transition to the productive state. Mean consecutive errors were higher for slow recoveries (0.211) than for fast recoveries (0.096), while mean detector confidence was higher for fast recoveries (0.824) than for slow recoveries (0.760).

**Figure 11:**
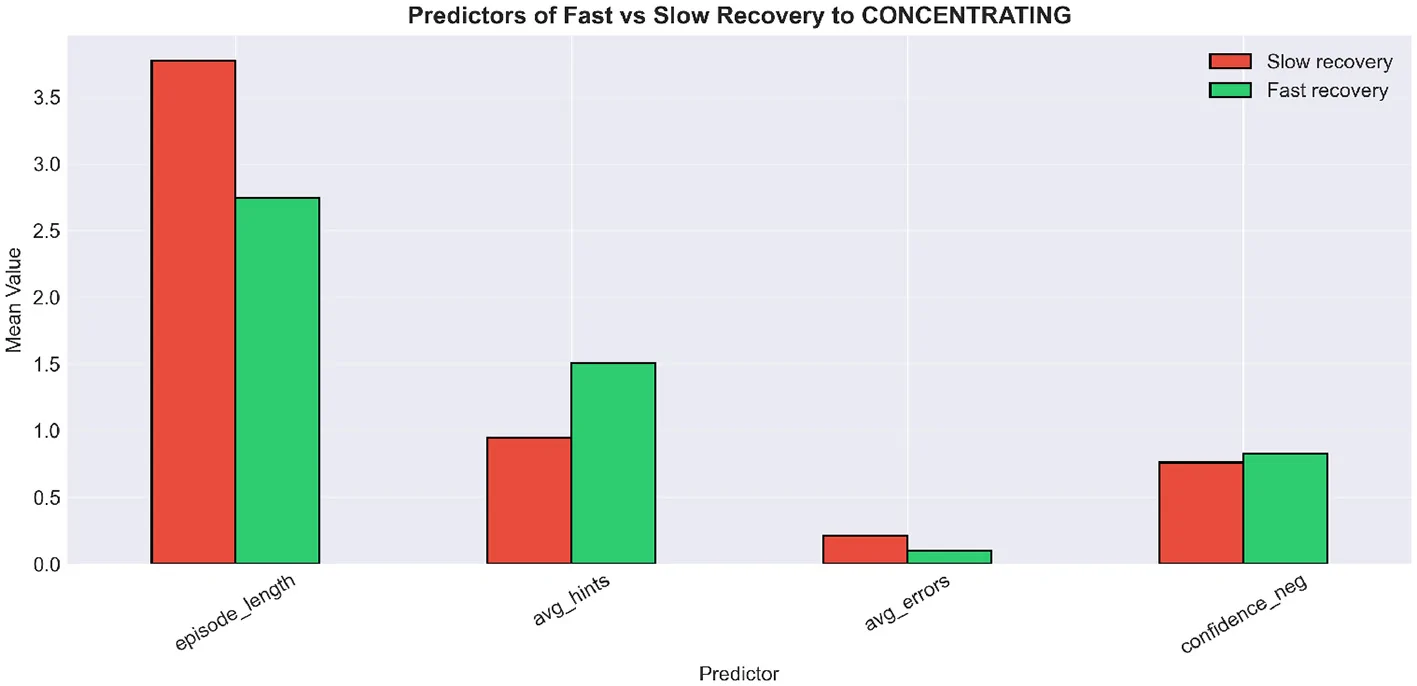
Mean predictor values for fast and slow cognitive-affective state recovery to CONCENTRATING.

Statistical validation of the differences between groups using the Kruskal–Wallis test with Bonferroni correction (Kruskal and Wallis, 1952; Dunn, 1961) confirmed the significance of all four predictors. With four simultaneous comparisons, the adjusted threshold was α^*^ = 0.050/4 = 0.0125, the same correction scheme applied in Section 4.3; all four predictors remained significant at *p* < 0.001, well below this threshold. Effect sizes η^2^ ranged from small to moderate (Tomczak and Tomczak, 2014): mean detector confidence registered the largest effect in the set (η^2^ = 0.040), followed by mean hint use (η^2^ = 0.037), mean consecutive errors (η^2^ = 0.025), and episode length (η^2^ = 0.002). Detector confidence, however, is not a behavioral variable but a covariate reflecting the certainty of the affect detector in assigning the negative state; among the three genuinely behavioral predictors, mean hint use registered the largest effect, followed by mean consecutive errors, with episode length presenting a practically trivial effect size and the lowest discriminative power in the set.

The aggregate association between hint use and fast recovery reported above pools episodes across all four negative states, which could obscure state-specific heterogeneity. To test this directly, a multivariate logistic model was fitted with recovery speed (fast vs. slow) as the outcome and avg_hints, avg_errors, episode length, mean detector confidence, and originating state as covariates. Controlling for these factors, avg_hints remained a significant positive predictor of fast recovery (coef = 0.124, *p* < 0.001, odds ratio ≈1.13), and the originating state itself contributed substantially to the model (pseudo-*R*^2^ = 0.205), with GAMING carrying by far the largest coefficient (coef = 3.60) relative to the reference state.

Recovery episodes were additionally classified as fast or slow using the median split of episode length (number of interactions) rather than recovery time in seconds, to test whether clock-time recovery is mechanically coupled to the originating state's item pace. Agreement between the two definitions of “fast recovery” was only 48.9%, close to chance level, confirming that the two metrics capture substantially different aspects of recovery. This divergence is most pronounced for GAMING, whose short item durations mechanically produce fast recovery in clock time regardless of how many interactions the episode contains, underscoring that clock-time and interaction-count recovery should not be treated as interchangeable outcomes. Combined with the state-dependent reversal of the hint-use association reported below, this indicates that “fast recovery” is not a single well-defined construct in this dataset, but one whose meaning shifts depending on both the metric chosen and the originating state.

The multivariate model treats the 63,646 recovery episodes as independent observations, despite their nesting within 797 students. To assess this, the model was refitted with cluster-robust standard errors grouped by student. Standard errors increased moderately relative to the naive model (1.68-fold for avg_hints), but every coefficient remained significant at *p* < 0.001, including avg_hints (coef = 0.124), indicating that the observed associations are not an artifact of treating within-student episodes as independent.

Table 8 reports the univariate association between hint use and fast recovery separately within each of the four negative states. The direction and significance of this association were not uniform: hint use was associated with faster recovery in BORED and FRUSTRATED episodes, consistent with the pooled result, but the association reversed in CONFUSED episodes, where greater hint use was associated with *slower* recovery, and was not statistically significant in GAMING episodes (*p* = 0.335), the most frequent negative state in the dataset. This pattern indicates that the facilitating role of hint-seeking identified in the pooled analysis is not a uniform behavioral mechanism, but is concentrated in specific states and absent or reversed in others.

**Table 8:** Hint use as a predictor of fast recovery, stratified by originating negative state.

State	Mean hints, fast	Mean hints, slow	η^2^	Direction
BORED	2.018	1.332	0.0162	Facilitating
FRUSTRATED	0.796	0.513	0.0139	Facilitating
CONFUSED	0.775	1.006	0.0147	Inverted
GAMING	2.825	2.802	< 0.001	Not significant (*p* = 0.335)

The stratified analysis was repeated on episodes reconstructed exclusively from interactions with an assignment margin of 0.10 or greater (42,298 of 63,646 episodes retained). The facilitating association for FRUSTRATED and the reversed association for CONFUSED were both replicated in this restricted sample (*p* < 0.001 in both cases), supporting the robustness of these two patterns. The BORED association, however, reversed direction under this stricter criterion (mean hints: 1.864 for fast recoveries vs. 2.255 for slow, η^2^ = 0.0082), indicating that its facilitating role in the full sample is not robust to the exclusion of low-margin assignments. The GAMING association became statistically significant under the larger restricted sample (*p* < 0.001) but with a negligible effect size (η^2^ = 0.0011), consistent with the interpretation that hint use carries no practically meaningful relationship with recovery speed in this state.

## 5 Discussion

The state prevalence structure replicates the hierarchy documented by Baker et al. (2010), with CONCENTRATING dominant and GAMING the most frequent form of disengagement, aligning with the patterns reported by Walonoski and Heffernan (2006) in the same environment. GAMING also exhibited the highest self-transition probability in the set (0.816), consistent with structural resistance to spontaneous recovery, while BORED showed the lowest (0.245) and was the only state where remaining in place was not the most likely transition, in line with its characterization by Andres et al. (2019) as a knowledge-level indicator rather than a sustained affective state. These self-transition probabilities were computed with self-transitions retained in the underlying frequency counts, following the corrected calculation recommended by Karumbaiah et al. (2019), who showed that excluding them biases the chance baseline against which transition probabilities are typically compared. The stationarity check, with a maximum difference of 0.045 across session thirds, supports treating transition probabilities as constant within a session, but this is distinct from the order of the model itself: the BIC comparison favored the second-order specification, indicating that the state two steps prior carries information the first-order model discards, concentrated around BORED, CONFUSED, and the CONCENTRATING-to-GAMING transition.

Recovery times differed substantially across states, from a median of 18.0 s for GAMING to 108.0 s for BORED, roughly a six-fold difference. The combination of high persistence with fast recovery in GAMING is only apparently contradictory: self-transition reflects the probability of remaining across consecutive items, not total episode duration, so the same rushed responding that sustains the state also produces a quick exit once it changes. FRUSTRATED and CONFUSED showed intermediate, similarly distributed recovery times, suggesting a partially shared resolution pattern between these two forms of active difficulty. BORED's combination of low persistence and long recovery time indicates that instability does not necessarily translate into speed: episodes may shift away from BORED quickly yet still take longer to reach CONCENTRATING specifically.

The convergence between Random Forest importance and Kruskal–Wallis effect sizes supports response time and hint use as robust discriminators, aligning with Baker et al. (2012) and Rahman et al. (2024). The two fine-selection criteria did not fully agree, however: RFECV excluded Total time on skill despite its individual importance exceeding threshold, showing that variable importance in isolation does not guarantee a non-redundant multivariate contribution. This divergence is itself informative, since it shows that the two methods can disagree even when the underlying signal is real, and that relying on a single selection criterion could have retained a redundant variable. The final four-variable set was nonetheless supported by both approaches, reinforcing confidence in its composition.

The positive association between GAMING and hint count in the multilevel model (+0.685 relative to BORED) is consistent with the established characterization of hint abuse as a core gaming behavior, where rapid, repeated hint requests bypass problem-solving rather than seek assistance (Baker et al., 2004b). Under this reading, elevated hint counts in GAMING episodes reflect exploitation of the scaffolding mechanism itself rather than the help-seeking behavior identified as facilitating recovery elsewhere. This distinction matters because it means hint frequency alone is an ambiguous signal without information on response latency or correctness. A system that treats every hint request as a sign of productive struggle would therefore misclassify a substantial share of GAMING episodes as genuine engagement.

The non-independence of interactions nested within students was addressed through a mixed-effects model with a random intercept per student. State-level differences in Time taken and Hint count remained significant at *p* < 0.001 after accounting for this structure, and the intraclass correlation coefficients were modest (ICC = 0.052 and 0.056), indicating that only about 5% of total variance is attributable to between-student differences. This does not eliminate the more general concern that large samples can yield statistically significant results of limited practical relevance (Sullivan and Feinn, 2012), but it does narrow the range of plausible explanations for the reported effects. The recovery model was subject to the same concern, given that recovery episodes are similarly nested within students; a cluster-robust refitting increased standard errors moderately (up to 1.68-fold) without altering the significance of any predictor, including hint use. Similarly, the dominance of timeTaken and hintCount does not resolve the circularity concern raised by their overlap with typical affect-detector inputs, since no independent, human-coded ground truth was available, and the sensitivity check narrows the range of plausible interpretations without settling the question; response time and hint-seeking remain the two signals on which any behavioral claim in this dataset chiefly rests.

Hint use was the strongest *behavioral* predictor of fast recovery in the pooled analysis (η^2^ = 0.037) and remained significant after controlling for originating state, episode length, and consecutive errors, though the state-stratified pattern is difficult to reconcile with a single mechanism. In line with the adaptive/maladaptive help-seeking distinction (Karabenick and Newman, 2009), hint requests plausibly function as adaptive help-seeking in BORED and FRUSTRATED, reflecting the self-regulatory decision described by Newman (1994); in CONFUSED, additional requests may instead indicate an avoidance-oriented or dependency-oriented profile; and in GAMING, hint use plausibly reflects exploitation of the scaffolding mechanism rather than genuine help-seeking of either kind. The cross-sectional design cannot distinguish these state-specific interpretations from a stable individual tendency to seek help that co-occurs differently with each state. This facilitating role, where it holds, should not be assumed to extend to learning outcomes: Schulz and Voermanek (2025) found that step-by-step hint abuse can undermine learning gains, suggesting recovery speed and learning may be distinct, occasionally competing objectives for intervention design.

A robustness check excluding low-margin assignments replicated the facilitating association for FRUSTRATED and the reversed association for CONFUSED, but reversed the BORED association itself, indicating that only the FRUSTRATED and CONFUSED patterns, along with their corresponding theoretical interpretations, can be considered robust to detector assignment uncertainty. This sensitivity is compounded by the divergence between the two recovery-time metrics reported above: since agreement between clock-time and interaction-count definitions of fast recovery was only 48.9%, the BORED-specific reversal itself may partly reflect which metric is used rather than a stable property of that state. Taken together, these two robustness checks suggest that conclusions about BORED specifically should be treated as provisional pending replication on data with higher-confidence state assignments and a clearer operational definition of recovery. The FRUSTRATED and CONFUSED patterns, by contrast, held under both forms of scrutiny and are the ones we consider best supported by the present analysis.

These results point toward a more differentiated use of state detection in ITS: rather than treating every negative state as an equally urgent trigger, systems could weigh a state's estimated recovery likelihood, pairing detection with a model of expected recovery trajectory instead of a standalone trigger. Bafandkar et al. (2025) describe PAPPL, a platform where a large language model (LLM) generates progressively targeted hints conditioned on attempt history and error patterns. The state-stratified findings reported here suggest such systems could also condition *whether* to surface a hint at all, prioritizing delivery in BORED and FRUSTRATED, withholding it by default in GAMING, and treating CONFUSED with particular caution given the reversed association observed. Embedding this kind of state-conditioned logic into LLM-based hint generation represents a direct extension of the present findings into currently deployed generative tutoring architectures.

The transition heterogeneity reported above adds a further qualification to the persistence patterns discussed earlier: while GAMING and BORED's self-transition probabilities differ in magnitude across performance terciles, their relative ordering does not, and GAMING remains the most persistent state regardless of student performance level. This suggests that the intervention priorities implied by structural persistence are broadly applicable across the performance spectrum, even though their urgency may vary somewhat by student profile, with lower-performing students showing both stickier negative states and weaker spontaneous recovery to CONCENTRATING. Aggregate transition matrices of the kind reported in Section 4.4 therefore remain informative for system-level design decisions, but a deployed intervention system could reasonably calibrate its thresholds using a coarse measure of student performance, prioritizing more proactive intervention for lower-performing students given their comparatively lower baseline recovery rates. This tercile-based comparison, following the approach used to contrast learning behavior patterns across performance groups in other ITS environments (Kinnebrew et al., 2013), cannot on its own determine whether the observed gradient reflects a causal effect of ability on recovery behavior or a shared underlying factor, such as prior domain knowledge, that independently shapes both performance and disengagement recovery.

The interpretation of these results is subject to several limitations. The argmax operationalization simplifies interactions with low assignment margins, and detector uncertainty was not modeled in the distribution and dynamics analyses. Additionally, the external validity of the underlying sensor-free detector itself (its accuracy against independent BROMP observations) was not re-evaluated in this study; the analysis relies on the detector's published validation from prior work (Baker et al., 2010), and any systematic bias in the original detector would propagate into every downstream result reported here. This uncertainty is not evenly distributed across states: BORED consistently showed the largest sensitivity to margin-based filtering across the prevalence, transition, and recovery-stratification robustness checks reported above, indicating that findings specific to BORED should be interpreted with particular caution relative to the other five states.

The Markov framework itself models only observable state transitions and cannot represent latent cognitive or affective processes that may underlie them; a Hidden Markov Model would allow such latent states to be estimated directly, and represents a natural extension of the order comparison reported above. Relatedly, the exact timing of a transition within the interval between two logged interactions is not observable in this dataset, so the analysis cannot determine whether a given transition occurred early or late within that window, nor whether transition likelihood depends on elapsed time since the previous state change. The recovery predictors identified are correlational, not causal, and greater hint use in fast recoveries could reflect either a facilitating effect or a stable student characteristic. The 30-min session boundary and 1,800-s recovery window, though consistent with standard practice (Kovanović et al., 2015), are threshold choices that could shift specific estimates. Finally, although the constructs examined here are argued above to generalize across interface generations, the analysis is confined to a single platform and a 2004–2007 dataset; the specific numeric estimates have not been validated on contemporary ITS platforms, and Paquette and Baker (2017) found disengagement patterns in ASSISTments to be partly environment-dependent, underscoring that generalization warrants direct testing rather than assumption.

## 6 Conclusions

The distribution of cognitive-affective states revealed a stable hierarchical structure in which CONCENTRATING was clearly the dominant state, while non-productive states were heterogeneously distributed with prevalences differing by more than an order of magnitude among themselves. This heterogeneity in prevalence does not translate into an equivalent heterogeneity in performance impact: BORED, the least frequent state, registered the lowest correct-response rate in the set, establishing a dissociation between frequency and severity that intervention models based exclusively on prevalence systematically underestimate. GAMING, by contrast, concentrated the largest share of disengagement in the sample, positioning it as the most consequential negative state by sheer volume even though its severity profile differed from that of BORED. This structure implies that the prioritization of adaptive interventions cannot rely solely on state frequency but must combine prevalence, persistence, and learning impact.

Transition modeling confirmed that stability is a graded, not binary, property across states. GAMING and CONCENTRATING were the most stable in the system, while BORED constituted the structural exception as the only state for which remaining in place was not the most probable transition. The structural resistance of GAMING to spontaneous recovery, combined with its high prevalence, positions it as the state of greatest practical relevance for the short-term design of adaptive interventions, whereas BORED's instability suggests that boredom episodes persisting long enough to require active intervention represent a particularly severe minority within that state. Both profiles underscore that a uniform intervention strategy is insufficient given this structural heterogeneity, and that persistence alone is a poor proxy for how urgently a given state warrants intervention.

Co-occurrence patterns among negative states in mixed episodes show differing frequencies. CONFUSED and FRUSTRATED formed the most frequent combination, followed by FRUSTRATED and GAMING, and then CONFUSED and GAMING, but these co-occurrence frequencies do not establish transition direction or systematic escalation. Further analysis of ordered transitions would be needed to identify potential early-intervention opportunities.

Variable selection identified four behavioral predictors with demonstrable discriminative power across states, with response time and hint use accounting for 81% of the total importance of the Random Forest model and the only effect sizes of large magnitude in the statistical validation. Item duration confirmed this hierarchy from an independent angle, with GAMING presenting by far the shortest duration in the set and OFFTASK and BORED the longest. This convergence across independent selection criteria, together with the circularity and robustness checks reported above, indicates that response pace and help-seeking behavior, rather than accumulated error history, are the most informative behavioral markers of cognitive state in this environment. Total time on skill was excluded from the final subset despite its individual importance, reinforcing that a variable's marginal contribution can be redundant once evaluated jointly with the rest of the set.

Recovery times differed substantially (roughly six-fold) across states, and this variation was not explained by persistence alone: GAMING resolved fastest despite being the most persistent state, while BORED, the least persistent, took longest to resolve. FRUSTRATED and CONFUSED occupied intermediate positions with similar medians, pointing to a partially shared resolution pattern between these two forms of active cognitive difficulty. The near-symmetric split between fast and slow recoveries around the global median further indicates that the classification threshold captures a genuine division in the data rather than an artifact of the cutoff itself. This dissociation between persistence and recovery speed indicates that state stability and recovery speed are distinct properties that intervention design should not conflate.

Hint use during the negative episode emerged as the strongest behavioral predictor of fast recovery in the pooled analysis, and this association held after controlling for originating state and episode characteristics in a multivariate model. State-stratified analysis, however, showed that this facilitating role is not uniform: it held in boredom and frustration episodes, reversed in confusion episodes, and was absent in gaming episodes, the platform's most frequent form of disengagement. Episode length, although statistically significant, carried a trivial effect size, ruling out disengagement depth as a useful indicator of recovery time in its own right. Together, these results indicate that hint frequency is a state-dependent signal rather than a general-purpose lever for recovery, and that the behavioral quality of an episode matters more than its extent.

The most direct line of future work is the implementation of this analytical pipeline within a purpose-built intelligent tutoring system operating in real classroom contexts with longitudinal tracking across full semesters, recording contextual variables from the physical classroom alongside ITS interactions. Integrating multimodal signals available in contemporary environments, such as browsing behavior, writing patterns, or camera-estimated attention metrics, would extend the identified behavioral predictors with variables of higher temporal resolution. Longitudinal tracking across semesters would further allow examining whether recovery patterns are stable within a student over time and whether they predict academic performance trajectories beyond the immediate interaction. Such a system would connect the cognitive recovery dynamics identified here to the long-term learning trajectories already documented by Pardos et al. (2014) and Ocumpaugh et al. (2016).

Cognitive recovery from disengagement episodes constitutes a phenomenon analytically distinguishable from state detection, with its own behavioral predictors that do not emerge from existing classification models. Active hint-seeking offers a concrete design principle for adaptive ITS, provided it is deployed selectively rather than as a uniform trigger across all forms of disengagement, a conclusion not supported by the present findings. The reversed and null associations found in confusion and gaming episodes, respectively, indicate that state-specific calibration is not optional but a precondition for any intervention built on this behavioral signal. Operationalizing these state-specific principles in systems with longitudinal tracking in real classroom contexts represents the most direct path toward translating these findings into measurable learning improvements.

## Data Availability

Publicly available datasets were analyzed in this study. This data can be found here: https://github.com/Jossgarciao/cognitive-recovery-assistments.
